# Supplementary material for: A genome-wide CRISPR screen identifies WDFY3 as a regulator of macrophage efferocytosis
Source: Nat Commun. 2022 Dec 24;13:7929. doi: 10.1038/s41467-022-35604-8 (PMC9789999; doi:10.1038/s41467-022-35604-8)
Supplement: Supplementary file 1 — Supplementary Information [file 41467_2022_35604_MOESM1_ESM.pdf]

Supplementary Information

A Genome-wide CRISPR Screen Identifies WDFY3 as a Regulator of Macrophage Efferocytosis

Jianting Shi, Xun Wu et al.

Supplementary Figures 1-11 ……………………..…………………..…………………………,……..…2

Supplementary Tables 1-14 ………………………………………………………………………………..17
1. Cell Lines and Primary Cells ………………………………………………………………………... 17
2. Mice ………………………………………………………………………………………..…………..…..18
3. gRNAs for CRISPR Screen Validation ……………………………………………..……………....19
4. Plasmids ………………………………………...………………………………………..……………....20
5. Primers for Genotyping ……………………………………………………………………..……….....21
6. Primers for Quantitative RT-PCR……………………………………………………………..……...…22
7. siRNAs …………………………………………………………………………………………...……..….23
8. Antibodies ……………………………………………………………………..………….……………….24
9. Cell Culture Medium …………………………………………………………………..…..…..…..….25
10. Chemicals and Recombinant Cytokines …………………………………………..……...…..26
11. Commercial Assay Kits ………………………………………………………………..…...…....27
12. Reagents for Efferocytosis and Phagocytosis Assays ……….……..………………...….……......28
13. Other Reagents and Supplies ……………………………………………..……………..…...…......29
14. Software and Algorithms ……………………………………………………………….…..…….….30

Supplementary Notes 1-3…………………………………………………………..…………….……31
1. Quantification of binding assay………………………………………………………………..…….…31
2. Quantification of F-actin ring …………………………………………………………..……..……..…32
3. Quantification of non-fragmented ACs in BMDMs and HMDMs ………………………..……….…33

Supplementary References ……………………………………………………………………..…...…..34

Supplementary Figures and Figure Legends

Supplementary Fig. 1

a

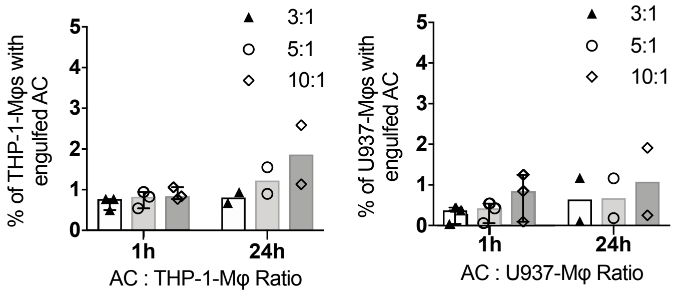

b

	Day 0 BM Cells	Day 6 After Selection	Transduction Rate (Day 6/Day 0 x 100%)	Day 9 BMDM	Before Efferocytosis	Sorted Non-Eaters	Sorted Good Eaters
Replicate 1	400 x 10^6	138 x 10^6	34.5%	140 x 10^6	40 x 10^6	15.0 x 10^6	2.5 x 10^6
Replicate 2	500 x 10^6	126 x 10^6	25.2%	130 x 10^6	34 x 10^6	16.6 x 10^6	3.5 x 10^6

c **Correlation of the two CRISPR screen replicates**

d

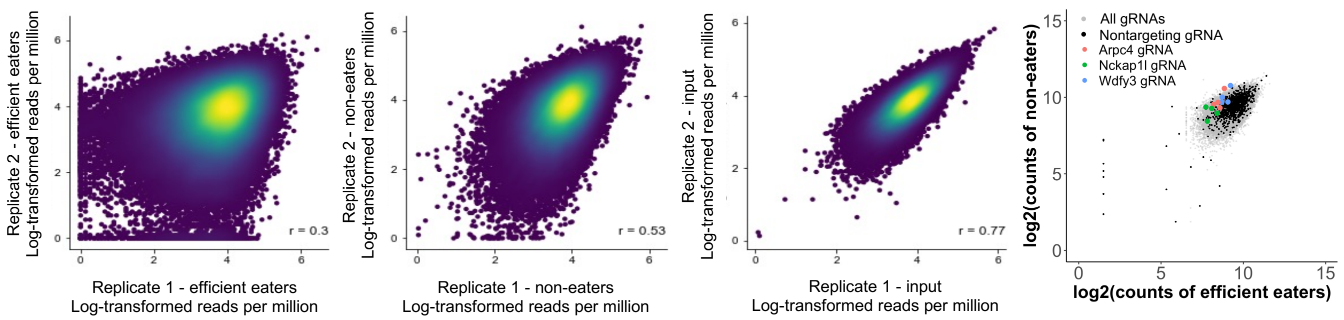

e

f

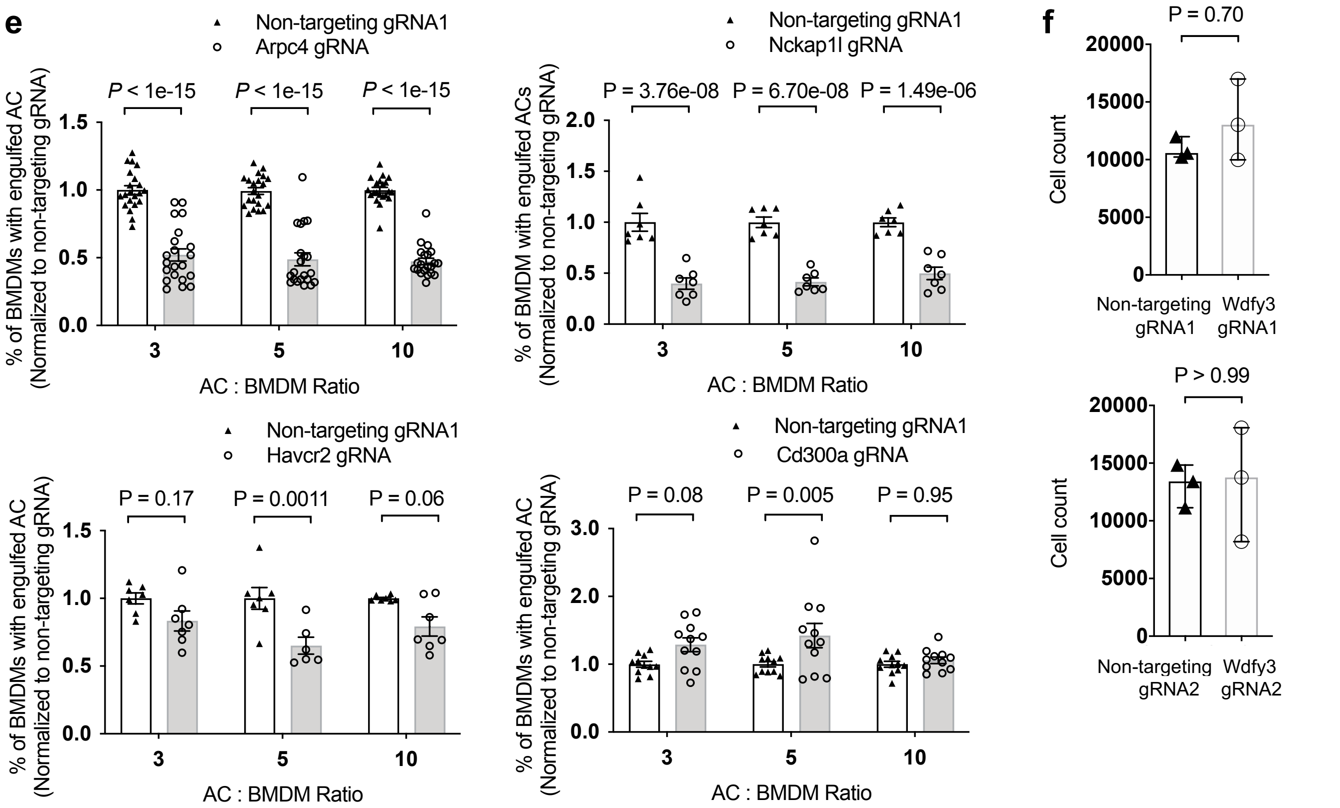

Supplementary Fig. 1 Quality control of our genome-wide CRISPR screen and validation of selected top hits.

(a) Efferocytosis of PKH26-labeled human Jurkat apoptotic cells by U937-derived macrophages and THP-1-derived macrophages (n = 2 or 3 independent experiments. The height of the bars represents median. The error bars represent 95% CI.) **(b)** The number of cells in each step for each of the two replicates of CRISPR screens. **(c)** Pearson's correlation coefficient for gRNA counts between the two replicates for "efficient eaters", "non-eaters", and the "input" samples. **(d)** Visualization of gRNA counts of all gRNAs, the non-targeting gRNAs, and the gRNAs targeting the top positive regulators. **(e)** High Content Imaging-based individual validation of top screen hits using gRNAs in the original screening library (n = 7 independent experiments for *Arpc4*, n = 3 independent experiments for *Cd300a*, n = 2 independent experiments for *Nckap1l* and *Havcr2*, each from the average of 3-5 technical replicates. Technical replicates were obtained from pooled cells from multiple biological replicates). **(f)** Knockout by gRNAs targeting *Wdfy3* did not affect BMDM viability (n = 3 independent experiments for each gRNA). Data are presented as mean ± SEM in panel **e** or median ± 95% CI in panel **f**. Two-sided P values were determined by a two-way ANOVA with Tukey's multiple comparisons test in panel **e**, or by Mann-Whitney test in panel **f**. AC, apoptotic cell; Mφ, macrophages.

Supplementary Fig. 2

Source:	This study	Haney et al., 2018								Kamber et al., 2021
Analysis Package:	MAGeCK	casTLE								casTLE
Sorting method:	FACS-based sorting	Magnetic beads-based sorting								FACS-based sorting
Screen library:	Brie Library	Customed genome-wide CRISPR/Cas9 deletion library (Morgens et al., 2017)								Customed genome-wide CRISPR/Cas9 deletion library (Morgens et al., 2017)
Phagocyte:	BMDMs	U937-derived macrophages								J774 macrophages
Substrates:	Apoptotic Jurkat cells (~ 10 µm)	IgG-opsonized RBCs (~ 4 µm)	C3b-opsonized RBCs (~ 4 µm)	Beads (4 µm, negatively charged)	Beads (1.3 µm, negatively charged)	Beads (0.3 µm, negatively charged)	Beads (1.3 µm, positively charged)	Zymosan (~ 1 µm)	Myelin (500 nm)	APMAP^{KO} Ramos cells (~ 10 µm)
Rank										
1	U2AF1	PRKCD	NCKAP1L	NHLRC2	NHLRC2	NHLRC2	NHLRC2	MAPK1	MAPK1	FERMT3
2	ARPC4	NCKAP1L	PRKCD	TM2D2	NCKAP1L	TM2D2	ACTR2	PRKCD	ITGB2	ITGB2
3	DYRK1A	PRKCB	MAPK1	LCMT1	ABI1	TM2D3	NCKAP1L	NHLRC2	NHLRC2	TLN1
4	WDR62	ABI1	ABI1	SYS1-DBNDD2	DOCK2	NCKAP1L	ACTR3	TLN1	TLN1	NCKAP1L
5	POMT2	MAPK1	DOCK2	TLE3	CD93	ARPC4-TTLL3	ABI1	PLEK	PRKD2	HSP90B1
6	PREX2	PRKD2	BRK1	TM2D3	CYFIP1	LCMT1	DOCK2	PRKD2	PRKCB	WASF2
7	HAVCR2	DOCK2	CREBBP	ABI1	LCMT1	TM2D1	CYFIP1	ITGB2	PRKCD	OTUD5
8	BCL2L13	AP002348.1	FBXO11	PTPN7	ACTR2	PPME1	ARPC2	FERMT3	SHOC2	BRK1
9	TRAF3	IKBIP	PRKCB	RPS6KA1	BRK1	RPS6KA1	ARPC4-TTLL3	PRKCB	FERMT3	GPR84
10	WDFY3	TCP1	CYFIP1	STUB1	STUB1	CHIC2	ARPC4	CERS2	SUCNR1	ITGAM
11	VPS52	SGMS1	SHOC2	NCKAP1L	ARPC2	KAT6A	SPPL3	NCKAP1L	PLEK	CNOT2
12	SEC61A1	MTDH	NPRL2	KAT6A	RP11-45M22.4	HSD17B12	LCMT1	SHOC2	RP11-45M22.4	ARPC4
13	KPNA6	MOBP	JUNB	OTUB1	TM2D2	DDA1	RAC1	SUCNR1	NCKAP1L	MESDC2
14	NCKAP1L	BRK1	AHSA1	TBL1XR1	PPME1	ABI1	ARPC3	ABI1	ARAF	RAB5C
15	FCRL1	FBXW12	PIK3R5	PPME1	WASF2	CYFIP1	MAPK1	JUNB	MEF2D	ARPC1B
16	VARS1	ZNF286B	AMBRA1	ARFRP1	LAMTOR4	HSPA13	AMBRA1	FADD	ABI1	UBR5
17	CEBPB	TMEM256-PLSCR3	DEPDC5	SYS1	ARPC3	STUB1	MYO9B	ELOVL1	JUNB	COMMD2
18	TMEM200A	CREBBP	SGMS1	UBE2D3	ACTR3	CUL3	RPL21	RIT1	ARPC4-TTLL3	MEMO1
19	ACTR3	NPRL2	ELOVL1	JMJD6	ARF1	UBE2D3	AIP	RP11-45M22.4	MESDC2	ACTR2
20	SLC2A12	FIS1	TLN1	XPR1	PPP2R1A	CD93	FADD	DOCK2	C16ORF72	DEK
21	TSC22D2	PAOX	RAC1	ELAVL3	TM2D3	AFF2	JAK1	BRAP	ERP44	MAPK14
22	ABHD17A	ARPC4	NPRL3	USE1	ARPC4-TTLL3	ACTR3	WASF2	GAB1	ARPC3	ABI1
23	RAC1	NARG2	PRKD2	C1ORF43	LAMTOR2	IQCA1	RRAGA	ACTR3	RIT1	RABGGTA
24	PREX1	FAM27D1	SUCNR1	CYFIP1	WDR81	TLE3	KLF6	FBXO11	HSP90B1	EED
25	SH3GLB1	JUN	SRM	MTA2	SASH3	BRD9	TM2D1	STT3A	KRAS	ARHGAP30
26	SNX24	AC022498.1	E2F8	GAPDH	FNIP2	GFM1	BIN2	TRAPPC2L	JAK1	TMEM189
27	CCDC115	TIMM17A	MORF4L2	WAS	TM2D1	MLLT1	BRK1	ACTR5	AHSA1	FRYL
28	SETD1B	LENEP	CERS2	DOT1L	ACTB	SLC25A51	LAMTOR4	ARNT	CERS2	SMARCB1
29	ABCE1	YOD1	RIT1	CHIC2	SAP130	TMEM167A	RP11-45M22.4	JAK1	ELOVL1	UBAP2L
30	CYFIP1	ERP44	EZH1	MCM3	RRAGA	CAND1	TM2D2	CSF2RB	RC3H1	TFAP2A
31	PIAS1	CYFIP1	ERP44	PCNX	RAC1	UBE2L3	BRD2	ARPC2	RAF1	9130011E15RIK
32	DCLRE1A	ZNF22	ARPC4-TTLL3	TM2D1	PPP6R1	RPL28	XPR1	ERP44	GNAI2	CITED2
33	GIMAP4	BASP1	PLEK	C11ORF73	ARPC4	C11ORF73	PTDSS1	ACTR2	RRAGA	GM20431
34	CEBPA	YPEL5	SASH3	PTPRC	TMEM208	RFC3	LAMTOR2	CREBBP	ACTR2	BHLHE41
35	RTL8A	LOR	RPS6KA3	ANAPC2	SPTLC1	ARPC2	PIK3R5	AMBRA1	GAB1	ITGB1
36	PRAMEF6	MAP3K4	DYRK1A	CD93	LAMTOR3	CCM2	OTUD5	AHR	ACTR3	KDM6A
37	TAS2R16	TBC1D29	C16ORF72	FURIN	CERS2	NSMCE1	C1GALT1C1	BSG	ARPC4	NCSTN
38	ATP1B3	ECHDC3	SAMD7	MYO9B	JKAMP	ZMYND8	UBE2D3	LRRC8A	AMBRA1	NRAS
39	ST8SIA2	RBM4B	ARAF	LDLR	MYO9B	ARPC3	HRC	ARAF	UBLCP1	FBXW7
40	COMMD4	RAX2	WASF2	BCL11A	PCNX	NPEPPS	USP34	SPPL3	RPS6KA3	SOS1
41	SPTY2D1	ELOVL1	YPEL5	KCTD5	TIMMDC1	LBX1	GNAI2	PPP1CC	STT3A	CCM2
42	WASF2	TRIM41	WDR3	PPP6R1	NARS2	WDR36	BASP1	CYFIP1	RRAGC	METTL16
43	ABI1	AHSA1	STT3B	LZTR1	SPTSSA	LZTR1	WDR81	RRAGA	CSK	UCHL5
44	TREM2	ELOVL5	SETD1B	GFI1	BASP1	CDC42	ATAD2	ARPC3	MAP2K3	ST3GAL3
45	SHLD1	TRNP1	KDM1B	CEBPE	HOXD13	KAT7	SF3B1	KRAS	ACTB	ZMYM4
46	GTF3C1	GADD45B	LRRC8A	TBC1D10B	ARF6	FLI1	ZCCHC7	TRBV27	CREBBP	FBXO11
47	TLE4	GSTT2B	MDN1	VPS54	CAPN9	C15ORF53	PACS2	RPS6KA3	NIFK	SLC25A26
48	ISY1	MEP1A	SNRNP25	NF1	COX20	PCNX	HECTD1	SLC35B4	BRAP	RAB1
49	OR10K1	AL591684.1	HNRNPF	TMEM203	NGLY1	PPP2R2A	PDCL	DAZAP1	ACTR5	UGP2
50	CDK9	MRPL2	SND1	SPRED2	UBE2A	CHD2	BCL6	C19ORF25	LAMTOR2	ARPC2
51	MGAT1	CDKL1	RAF1	RAB10	MESDC2	PTPN7	MRPL18	QKI	RAB7A	YTHDF3
52	VPS33A	FKBP5	THRAP3	KIAA1109	UBE2D3	CAB39	TOR1B	TRAF6	OSTC	PSMA1
53	ACTR2	TRIM48	ARL4A	SLC38A2	CCER1	ANLN	C5ORF47	C16ORF72	DOCK2	KRAS
54	LACTB	FEZF2	PRKRA	PAXBP1	CHMP5	STK11	CDK11A	MEF2D	CYFIP1	YWHAE
55	FBXW12	FBXO11	KLF16	RPL28	C1GALT1C1	ARF4	TMEM30A	GNAI2	ZNF699	CYFIP1
56	ELOVL5	RAC1	ITFG2	ABT1	NFE2L2	TBCB	VPS4B	IRF8	ELF1	MLLT1
57	HNRNPK	ZNF699	ATAD1	PRELID1	LSMD1	AKT2	C1ORF220	APEH	BRK1	INTS10
58	SF3B2	SARNP	CTSE	NAPG	MAFA	UBE2K	EWSR1	MAP2K3	ARPC2	RUNX1
59	NFKBIA	FJX1	PAPD5	PRPH2	AC021860.1	RSRC2	SFTPA1	LARP4	YPEL5	SPOP
60	FBXW2	TIMM13	BIN2	SBDS	MAB21L3	AAGAB	ACAD8	AIP	SPCS2	ZKSCAN5
61	DENND3	RNF187	EHD3	AFF2	KIAA1432	TNFAIP3	TRAPPC2L	STIP1	MAGT1	SH3BP2
62	TMEM220	CKAP4	CARM1	BRK1	GPS2	ANAPC10	RAX	ARPC4-TTLL3	KSR1	WAS
63	CCDC9B	RAD51AP1	FERMT3	MYEOV2	MYBPC2	KCTD5	ZFAND4	CSK	BCL6	ELAVL1
64	ALPK1	ALKBH2	OR1S1	NUDCD2	TDRD3	MTA2	DNAJB4	UBL5	REC8	WIPF1
65	UROD	AKAP2	C12ORF66	COQ10B	RPL28	GDF1	ARPC5	JAK2	MAP2K1	FXR1
66	ZBTB46	AL592284.1	ZBTB1	NCOR1	FAM90A1	WI2-3308P17.2	RP11-146D12.2	GPR89C	ALK	BC051142
67	ODAD2	PKN3	PHF6	SRRD	USF1	CBWD7	CSTF3	CSF2RA	STT3B	PTPRO
68	SOCS5	RARRES2	LBX1	NSMCE2	TMEM60	HIST2H2AA4	CASP9	PIK3R5	SAMSN1	LRRC25
69	PDCD1	AC020922.1	STEAP3	TUSC1	PIK3R5	VPS11	WSB1	FLCN	GABARAPL2	OLFR1167
70	TAS1R1	ABHD14B	GRPEL1	CCNH	HIST1H2BJ	RPRD2	SASH3	RREB1	FOXJ3	PNRC1
71	FIZ1	AK2	FRG2C	CCNF	SERP1	TFEB	PDHA1	UTP15	RAX	DSTYK
72	GRIN2D	TP53	NXF5	TNPO1	AMBRA1	C1ORF43	KDM2A	BCL6	CELF2	CLASRP
73	TOPAZ1	C2ORF27B	KPTN	NIP7	SNRK	RAB1A	AKT1	KDM1B	CTTNBP2	CFP
74	ARPC3	RP11-204N11.1	RC3H1	JKAMP	HIST1H2AH	WDR7	FMO5	MS4A8	MSI1	XPO6
75	MTMR9	SULT1C2	SKA1	WASF2	NDUFA4	TMEM203	GOLGA8S	LAMTOR3	GSK3B	FLCN
76	TMEM98	NKD2	MAF1	ZMYND8	ELMO2	MYO9B	CERS2	FNIP2	OR2D3	SURF2
77	FBXO11	PYURF	KATNA1	LIMS3L	SERBP1	ACTR2	KLK14	DEPDC5	NDUFA4L2	SUZ12
78	CCDC88B	LCE1F	SPAM1	TVP23C-CDRT4	TFEB	AC009060.1	EHD1	OR4A16	FAM83A	WDR26
79	WNK1	CYS1	PTPLB	MIDN	C21ORF128	ZFR	PPP2R2A	RAB7A	IGSF9	TADA2B
80	CEACAM4	HSBP1L1	ITIH1	GTPBP3	TCERG1	ZC3HC1	PROCA1	AHSA1	FBXO11	RAB14
81	SERPINB3	TMEM11	ELF4	ZC3HC1	FERMT2	ZFX	PKN1	API5	SETD1B	CRAMP1L
82	OPA3	CDKN2C	ERG	FTO	CCNF	PA2G4	APBB3	AC025278.1	WTAP	PIK3CB
83	CTR9	DUS3L	ARGLU1	PDHA1	STT3A	MYC	PITRM1	BIN2	FGL1	MAFB
84	PRL	ZNF503	SFPQ	KIF11	TLN1	COX8A	ELOVL1	RP11-512M8.5	WASF2	ZBTB7A
85	U2AF2	AL645730.2	USP46	SAP130	ARFRP1	ELAVL1	BRAP	CBLC	LAMTOR4	ZFP644
86	METTL16	GORASP1	RNF133	EWSR1	HSP90B1	CSNK2A2	KIAA1109	SCD	PHACTR4	PIK3R1
87	POT1	PIK3R6	TRIM48	RAX	BRD7	PET112	USP17L2	RRAGC	SPPL3	DNTTIP1
88	APOL4	KCTD13	NHLRC2	XPO6	NOC2L	PEPD	CSTF2	FAM203A	CTNND2	ADRBK1
89	APOL1	GJA3	TECPR2	AC019206.1	LLPH	XPR1	PPIAL4D	PTPLB	LAMTOR3	CSE1L
90	APOL3	C5ORF20	BRD2	REG1A	WAS	LAMTOR2	SEC23B	GNB1	ZSWIM8	GNB2
91	APOL2	CYBB	DNAH5	FBXW12	NUDCD2	STAM	TRAF2	TECR	ICMT	PREX1
92	KATNBL1	MRPS18A	BRAP	RP11-763F8.1	TPRKB	RASA2	XRCC6BP1	STT3B	FNIP2	STRIP1
93	TDO2	NAA15	EIF5B	ELAVL1	DAXX	NUDCD2	SRRT	FOXJ3	BIN2	SNX17
94	RHO	AL359091.2	MAPK14	KIAA1432	APRT	CCNF	KIDINS220	GADD45B	CNOT11	RELA
95	THAP3	RAB13	CCDC6	NEDD8	WAPAL	PDHB	DDX28	TRAF3IP3	PLA2G4E	MAP4K4
96	NPY4R	AC112721.1	MUL1	WDR81	AUP1	CDC73	PGAM1	RIC8A	CDC42	DPH2
97	NPY4R2	JUNB	TCEANC	AMBRA1	DHTKD1	ZGLP1	MTHFD1	ELOVL5	ADRM1	MATK
98	LYG2	HIST1H1E	SZT2	SNRK	HSA-MIR-150	ACKR4	CDC42	SFPQ	BOD1	TMEM237
99	ATP6V0B	SUN1	CHMP3	UNC50	FLCN	COX6B1	CREBBP	ELF4	RASGRP3	ARPC3
100	RPS23	RNF38	MESDC2	CHMP6	HAUS7	HNRNPA2B1	RPSA	MYD88	SASH3	OLFR389

Frequency
1
2
3
4
5
6
7
8
9
10

Supplementary Fig. 2 Comparing the top-ranked positive regulators by our screen and previous screens in U937 monocytic cell line-derived macrophages or J774 macrophages using an array of substrates.

The heatmap visualizes the overlap of the top-ranked positive regulators in our screen of efferocytosis in primary macrophages and in previously published screens of phagocytosis of an array of substrates in U937-derived macrophages (Haney et al, 2018[1]) or J774 macrophages (Kamber et al, 2021[2]). The matrix for generating the heatmap is in **Supplementary Data 6**. Murine gene symbols were converted to human gene symbols using gProfiler at https://biit.cs.ut.ee/gprofiler/orth.

Supplementary Fig. 3

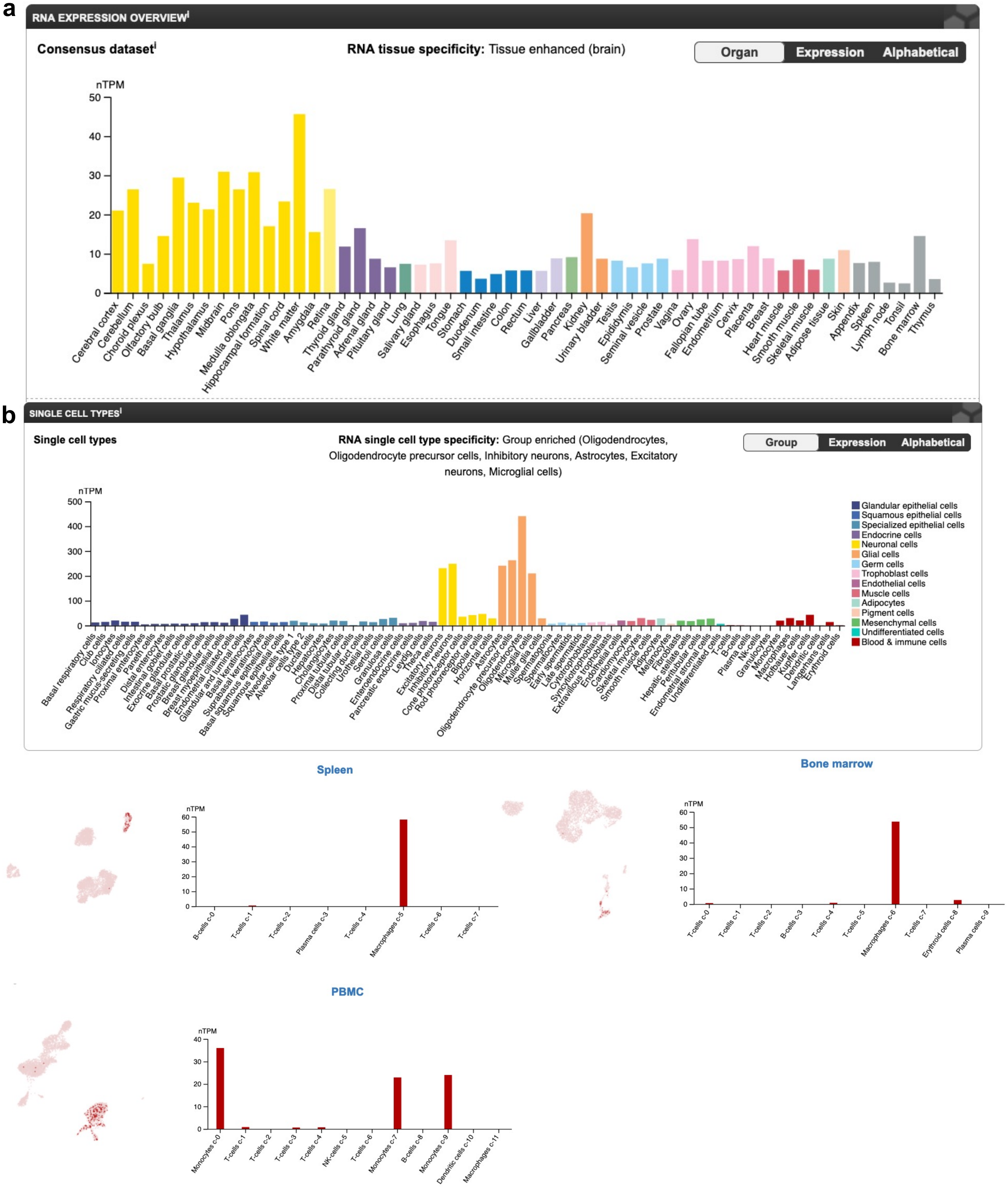

c

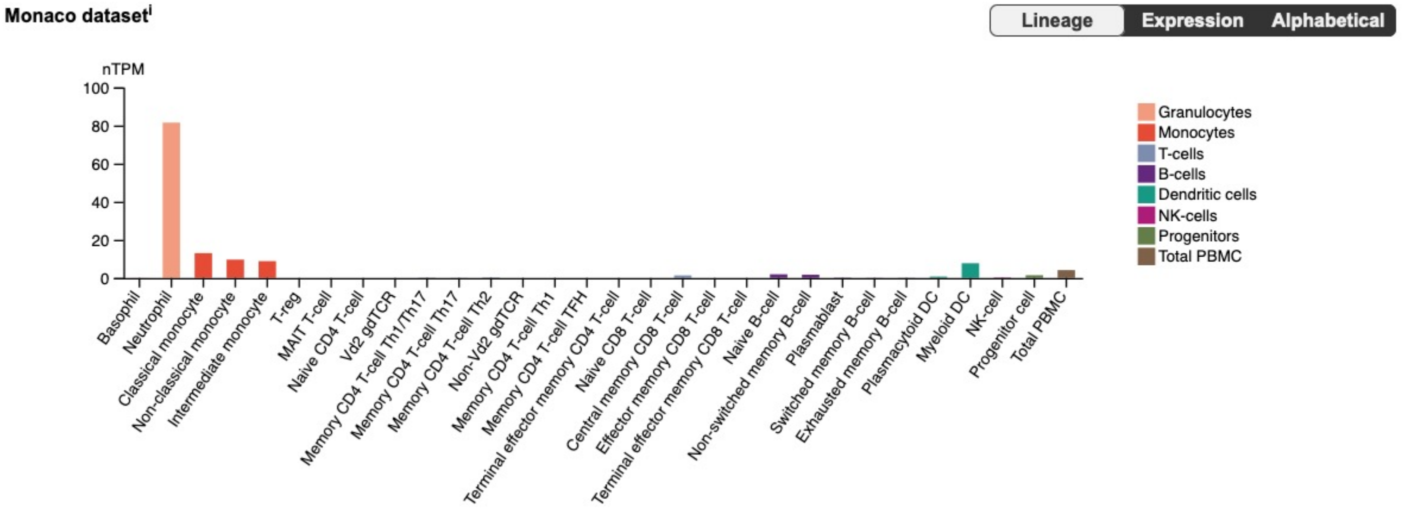

Supplementary Fig. 3 *WDFY3* expression across tissue and cell types based on data in the Human Protein Atlas resource.

(a) The consensus dataset consists of normalized transcript expression (nTPM) levels for 55 tissue types, created by combining the HPA and GTEx transcriptomics datasets using the internal normalization pipeline. Color-coding is based on tissue groups, each consisting of tissues with functional features in common. **(b)** Single cell transcriptomics data for 25 tissues and peripheral blood mononuclear cells (PBMCs) were analyzed. These datasets were respectively retrieved from the Single Cell Expression Atlas, the Human Cell Atlas, the Gene Expression Omnibus, the Allen Brain Map, and the European Genome-phenome Archive. **(c)** The nTPM levels resulting from the internal normalization pipeline are visualized for 29 blood cell types and total PBMCs from Monaco et al, 2019[3]. The panels are screenshots from the Human Protein Atlas resource on November 24, 2021.

Supplementary Fig. 4

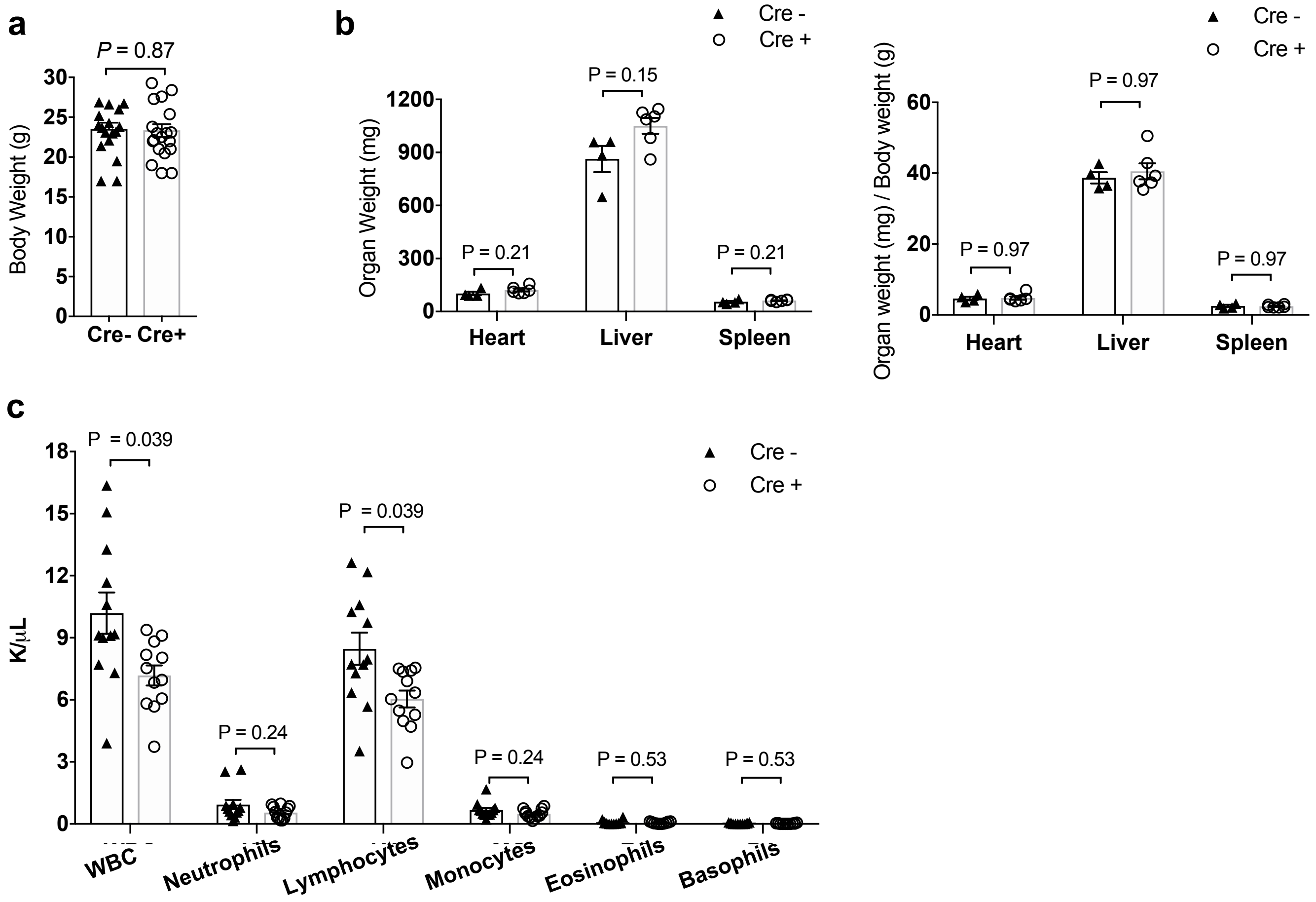

Supplementary Fig. 4 Basic characteristics of myeloid-specific *Wdfy3* knockout mice.

(a) Body weight (n = 18 and 20 biological replicates for Cre^- and Cre^+, respectively). **(b)** Organ weight and organ weight normalized to body weight (n = 4 and 6 biological replicates for Cre^- and Cre^+, respectively). **(c)** Complete Blood Count with Differential (n = 12 biological replicates). Data are presented as mean ± SEM. Two-sided P values were determined by unpaired t-test. WBC, white blood cells.

Supplementary Fig. 5

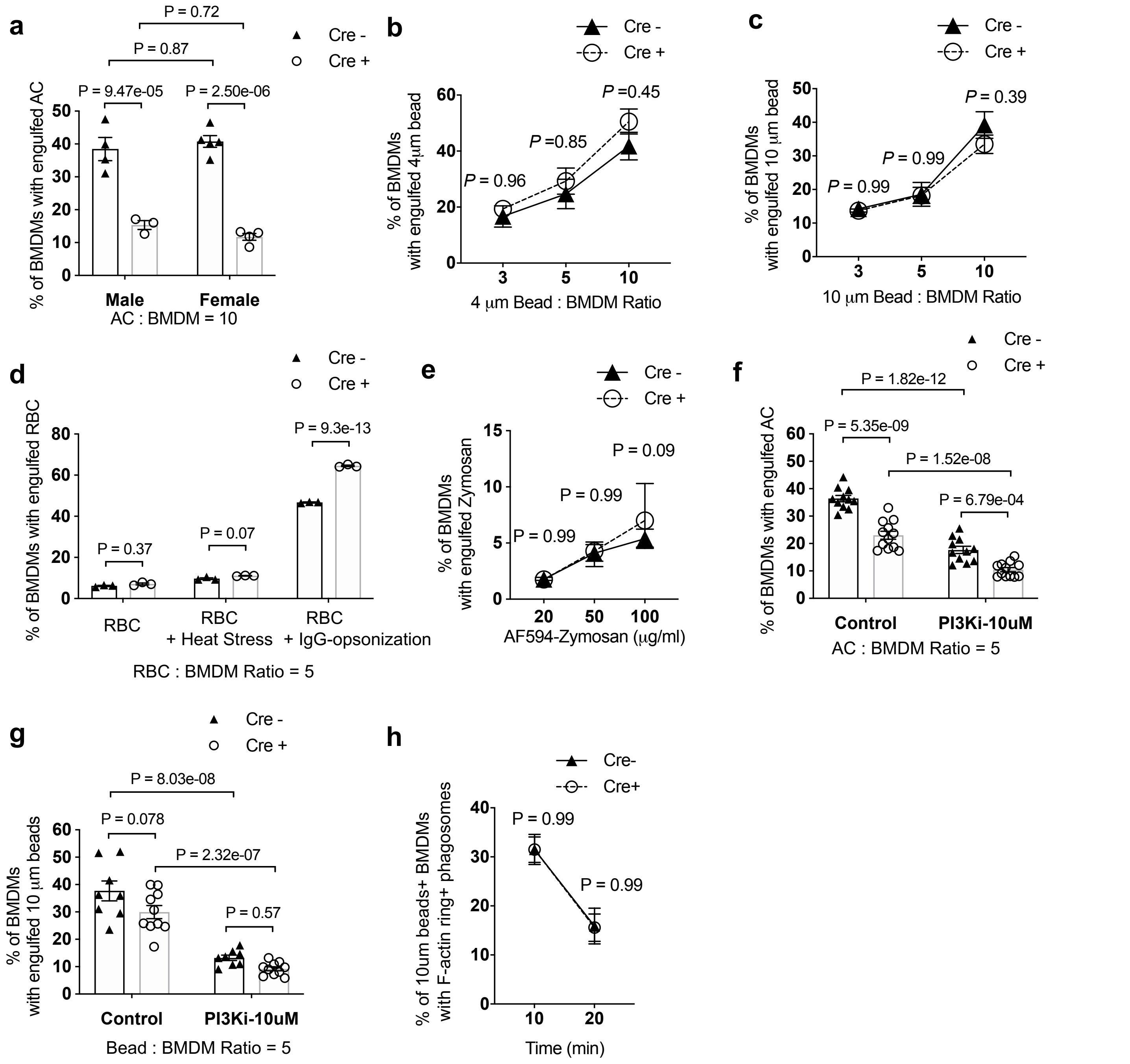

Supplementary Fig. 5 Phagocytosis of different substrates and the involvement of the PI3K pathway.

(a) Impaired uptake of ACs were confirmed in both male and female mice (n = 4 and 3 biological replicates each with 2 technical replicates for male mice, n = 5 and 4 biological replicates each with 2 technical replicates for female mice). **(b)** 4 μm latex beads (n = 3 biological replicates with the average of 2 technical replicates). **(c)** 10 μm beads (n = 3 biological replicates with the average of 2 technical replicates). **(d)** Sheep red blood cells (RBCs) were either untreated, heat-stressed, or IgG-opsonized (n = 3 biological replicates with the average of 2 technical replicates). **(e)** Alexa Fluor (AF)-594 labeled Zymosan particles (n = 6 biological replicates with the average of 2

technical replicates). **(f)** Uptake of ACs by BMDMs with treatment of PI3K inhibitor, LY294002 (n = 11 and 12 biological replicates for Cre^- and Cre^+, respectively). **(g)** Uptake of 10 μm beads by BMDMs with treatment of PI3K inhibitor, LY294002 (n = 8 and 10 biological replicates for Cre^- and Cre^+, respectively). **(h)** BMDMs were stained with CellTracker Green and siR-actin, then incubated with red fluorescent beads for various time points (10 min and 20 min). For each time point, unbound beads were removed and BMDMs were fixed. BMDMs were imaged and the percentage of BMDMs with engulfed beads surrounded by F-actin rings in all BMDMs with engulfed beads was quantified (n = 6 biological replicates). Data are presented as mean ± SEM. Two-sided P values were determined by a two-way ANOVA with Tukey's multiple comparisons test.

Supplementary Fig. 6

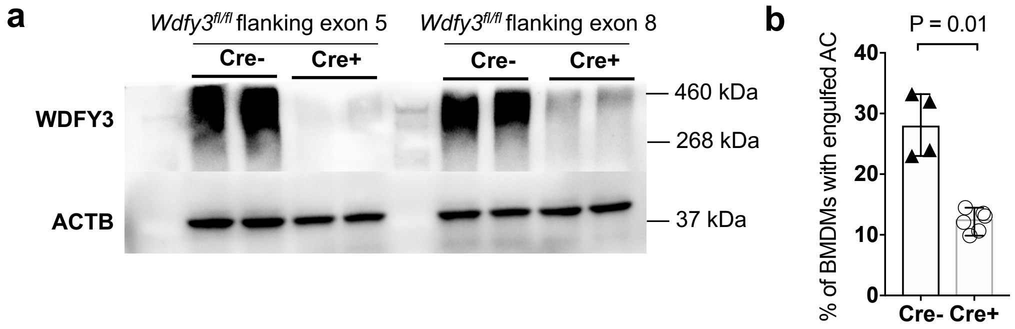

Supplementary Fig. 6 Validation of impaired efferocytosis in BMDMs from myeloid-specific *Wdfy3* knockout mice on C57BL/6NJ background.

(a) *Wdfy3*$^{fl/fl}$ mice generated by the Knock-Out Mouse Project (KOMP) with two *loxP* sites flanking exon 8, were maintained on C57BL/6N background[2]. Breeding to *LysMCre* mice led to efficient knockout of *Wdfy3* though a small amount of residual protein remained detectable (n = 2 biological replicate). **(b)** Uptake of Hoechst-labeled ACs was impaired in BMDMs of Cre^{+} mice with Cre-lox mediated deletion of exon 8 and on C57BL/6NJ background, supporting the role of *Wdfy3* in macrophage efferocytosis independent of the genetic strain and the specific gene-inactivating mutation of the mouse models (n = 4 and 6 biological replicates for Cre^{-} and Cre^{+}, respectively with the average of 2 technical replicates). Data are presented as median ± 95% CI. Two-sided P values were determined by Mann-Whitney test.

Supplementary Fig. 7

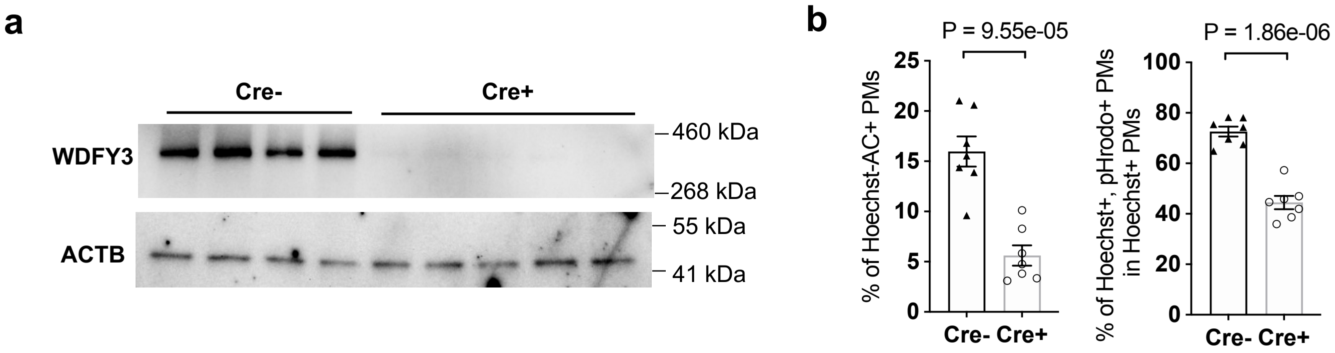

Supplementary Fig. 7 Validation of knockout, and impaired uptake and acidification in peritoneal macrophages (PMs) of Cre^{+} *Wdfy3* knockout mice.

(a) Validation of efficient knockout of *Wdfy3* in PMs by western blot (n = 4 and 5 biological replicates for Cre^{-} and Cre^{+}, respectively as shown in the representative blot)**. (b)** Cre^{-} and Cre^{+} PMs were incubated with ACs labeled by Hoechst, which stains DNA and is pH-insensitive, and pHrodo, which is pH-sensitive and shows fluorescent signal only under an acidified environment in the phagolysosome. The percentage of Hoechst^{+} PMs indicates uptake. The percentage of Hoechst^{+}/pHrodo^{+} PMs in Hoechst^{+} PMs indicates acidification of the engulfed cargos (n = 7 biological replicates, each from the average of 2 technical replicates). Data are presented as mean ± SEM. Two-sided P values were determined by unpaired t-test.

Supplementary Fig. 8

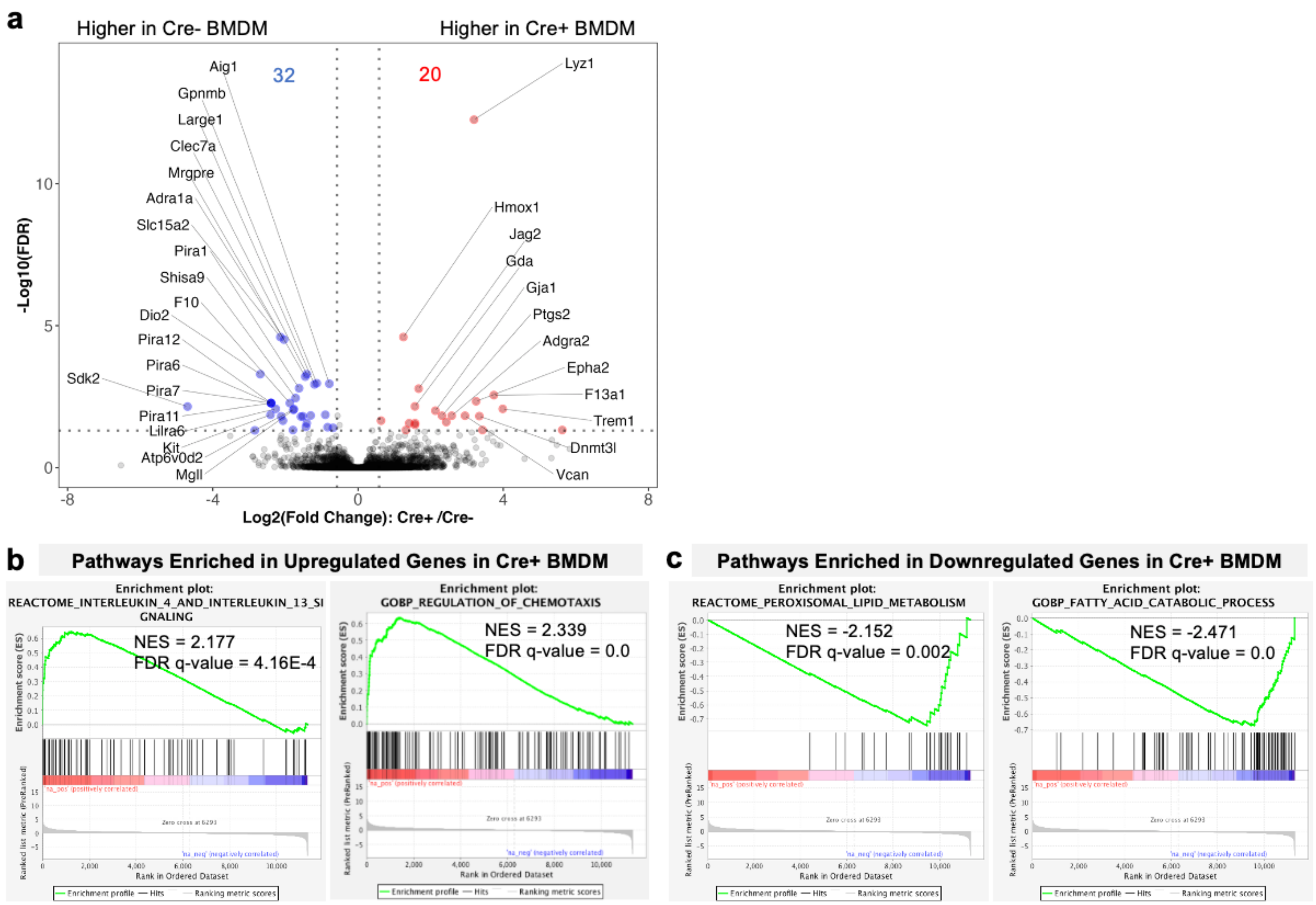

Supplementary Fig. 8 The effects of *Wdfy3* deficiency on BMDM transcriptomic profile by RNA-seq.

(a) Volcano plot highlights differentially expressed (DE) genes in Cre^+ vs. Cre^- BMDMs (n = 4 biological replicates, male mice, the DESeq2 output is shown in **Supplementary Data 7**. **(b)** Selected top Human Reactome Pathway and Gene Ontology Biological Process terms enriched in the upregulated genes in Cre^+ BMDMs vs. Cre^- BMDMs. Complete GSEA results are shown in **Supplementary Data 8** and **Supplementary Data 9**. **(c)** Selected top Human Reactome Pathway and Gene Ontology Biological Process terms enriched in the downregulated genes in Cre^+ BMDMs vs. Cre^- BMDMs. Complete GSEA results are shown in **Supplementary Data 10** and **Supplementary Data 11**.

Supplementary Fig. 9

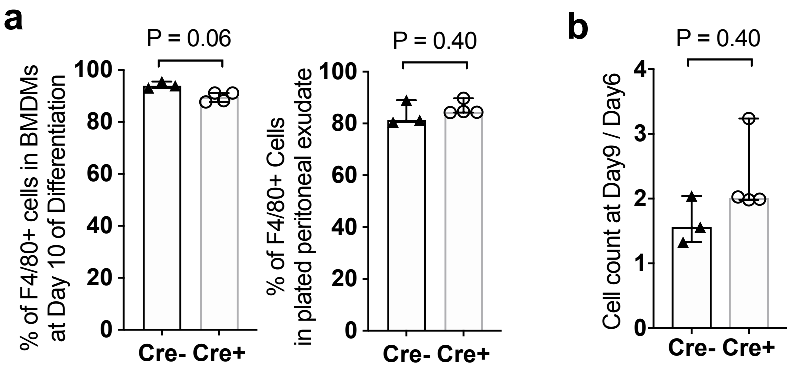

Supplementary Fig. 9 *Wdfy3* knockout did not affect macrophage differentiation and proliferation.
(a) The percentage of F4/80^{+} macrophages in BMDMs and peritoneal exudate was comparable between Cre^{-} and Cre^{+} mice (n = 3 and 4 biological replicates for Cre^{-} and Cre^{+}, respectively with an average of 2 technical replicates). **(b)** Bone marrow **(**BM) cells were differentiated to BMDMs in 9 days and cell numbers were counted on day 6 and day 9. The ratio of cell counts on day 9 / day 6 implicates population doubling, indicative of proliferation capacity. The ratio shows no statistical difference between Cre^{-} and Cre^{+} mice (n = 3 and 4 biological replicates for Cre^{-} and Cre^{+}, respectively). Data are presented as median ± 95% CI. Two-sided P values were determined by Mann-Whitney test.

Supplementary Fig. 10

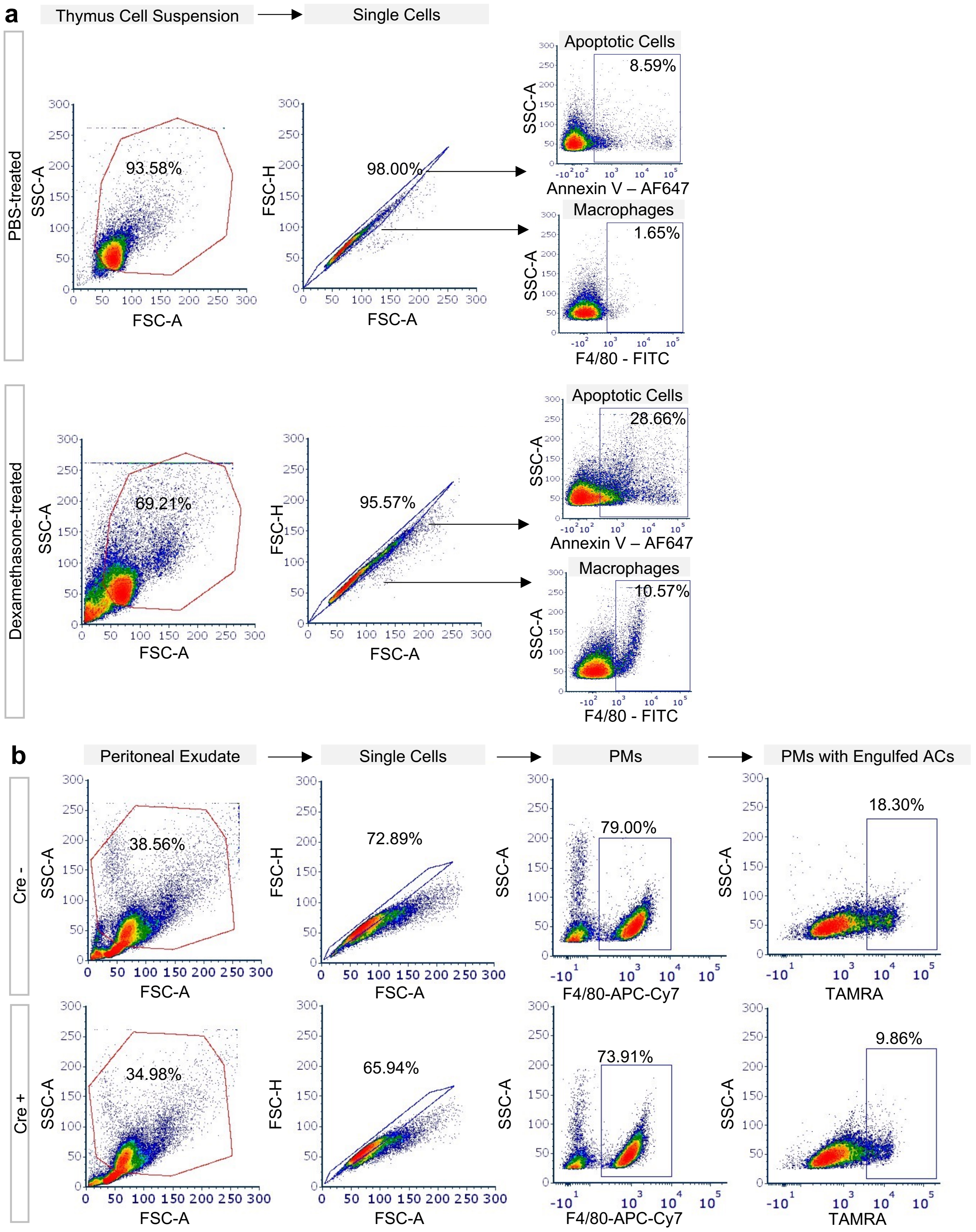

Supplementary Fig. 10 Gating strategy of flow cytometry analysis.

(a) Gating strategy of flow cytometry analysis for *in vivo* thymus efferocytosis. **(b)** Gating strategy of flow cytometry analysis for *in vivo* peritoneal macrophage efferocytosis.

Supplementary Fig. 11

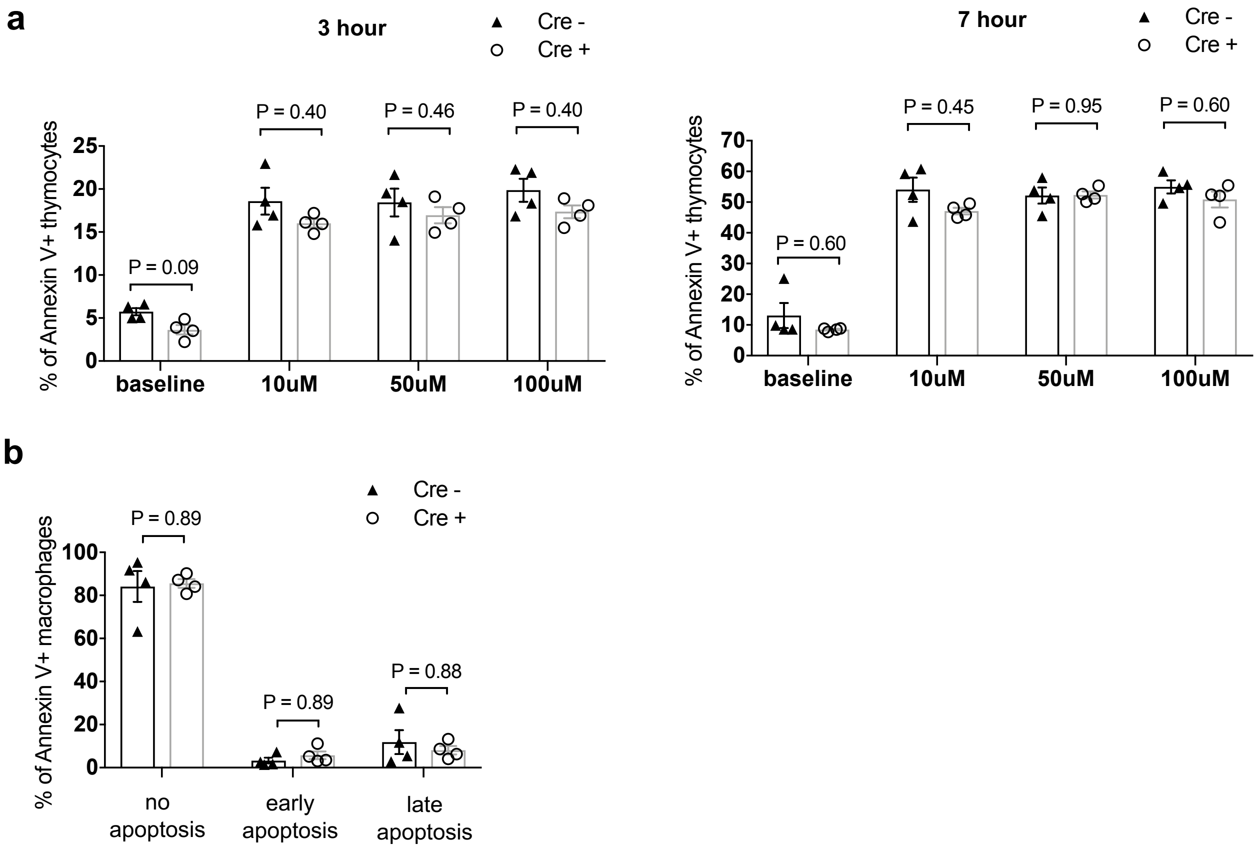

Supplementary Fig. 11 Myeloid-specific *Wdfy3* knockout did not impact the apoptosis rate induced by dexamethasone in thymocytes and BMDMs.

(a) Thymocytes were isolated from Cre^- and Cre^+ mice and cultured in DMEM media supplemented with 10% FBS at 37 °C, 5% CO_2 with or without dexamethasone treatment at the indicated concentration. The % of Annexin V+ thymocytes was determined at 3 h and 7 h (n = 4 biological replicates). **(b)** BMDMs were treated with 100 µM dexamethasone in DMEM media supplemented with 10% FBS at 37 °C, 5% CO_2 for 7 h, and the % of Annexin V+ BMDMs was determined (n = 4 biological replicates). Data are presented as mean ± SEM. Two-sided P values were determined by a two-way ANOVA with Tukey's multiple comparisons test.

Supplementary Tables

Supplementary Table 1. Cell Lines and Primary Cells

Reagent or Resource	Source	Identifier
Human: Jurkat cells	ATCC	TIB-152
Human: THP-1 cells	ATCC	TIB-202
Human: U937 cells	ATCC	CRL-1593.2
Human: Peripheral Blood Mononuclear Cells	New York Blood Center	N/A
Mouse: L-929 Fibroblasts	ATCC	CCL-1
Mouse: Bone Marrow-Derived Macrophages	This paper	N/A
Mouse: Peritoneal Macrophages	This paper	N/A

Supplementary Table 2. Mice

Reagent or Resource	Source	Identifier
Wild-type: C57BL/6J	The Jackson Laboratory	JAX: 000664
GFP-LC3: C57BL/6J	Ai Yamamoto Lab, Eenjes et al., 2016.[4]<br>Kuma et al., 2008.[5]	N/A
LysMCre$^{+/-}$: C57BL/6J	The Jackson Laboratory	JAX: 004781
Rosa-Cas9 knockin: C57BL/6J	The Jackson Laboratory	JAX: 026179
Wdfy3$^{fl/fl}$: 129/SvEv x C57BL/6 (flanking Exon 6)	Ai Yamamoto Lab,<br>Dragich et al., 2016.[6]	N/A
Wdfy3$^{fl/fl}$: C57BL/6NJ (flanking Exon 8)	Konstantinos Zarbalis Lab,<br>Orosco, L. A. et al. 2014.[7]	N/A

Supplementary Table 3. gRNAs for CRISPR Screen Validation

Target	Source	gRNA sequence (5' to 3')	PAM	Target Context Sequence (5' to 3')
Arpc4 gRNA	Brie Library (Addgene Cat#73633)	CTTTGTAATCCACTTCATGG	AGG	TGGACTTTGTAATCCACTTCATGGAGGAGA
Cd300a gRNA	Brie Library (Addgene Cat#73633)	GGGAATAGTCATGTTCACGG	TGG	CCCTGGGAATAGTCATGTTCACGGTGGCCC
Havcr2 gRNA	Brie Library (Addgene Cat#73633)	CTAAAGGGCGATCTCAACAA	AGG	CCAGCTAAAGGGCGATCTCAACAAAGGAGA
Nckap1l gRNA	Brie Library (Addgene Cat#73633)	GACACGCTGGTATATGCCTG	TGG	CTTCGACACGCTGGTATATGCCTGTGGTTG
Non-targeting gRNA1	Brie Library (Addgene Cat#73633)	GGGGTAGGCCTAATTACGGA	N/A	N/A
Non-targeting gRNA2	Sigma-Aldrich (Cat# CRISPR18)	N/A	N/A	N/A
Wdfy3 gRNA1	Brie Library (Addgene Cat#73633)	ATGAAGTCTGATGTCATGAG	GGG	ACCCATGAAGTCTGATGTCATGAGGGGTCG
Wdfy3 gRNA2	Sigma-Aldrich	Refer to Sanger Clone ID# MM5000009433	N/A	CATGGTGACGGAGATCCGGAGG

Supplementary Table 4. Plasmids

Reagent or Resource	Source	Identifier
pcDNA-myc-WDFY3$_{2543\text{-}3526}$	Ai Yamamoto Lab,<br>Filimonenko et al., 2010.[8]	N/A
pDEST-tdTomato-WDFY3$_{2981\text{-}3526}$	Ai Yamamoto Lab,<br>Filimonenko et al., 2010.[8]	N/A
pLE4	Fang et al., 2018.[9]	N/A

Supplementary Table 5. Primers for Genotyping

Strain	Source	Forward (5' to 3')	Reverse (5' to 3')
LysMCre$^{-/-}$	The Jackson Laboratory	CCCAGAAATGCCAGATTACG	CTTGGGCTGCCAGAATTTCTC
LysMCre$^{+/-}$	The Jackson Laboratory	TTACAGTCGGCCAGGCTGAC	CTTGGGCTGCCAGAATTTCTC
Wdfy3$^{fl/fl}$: 129/SvEv x C57BL/6 (flanking Exon 6)	Ai Yamamoto Lab, Dragich et al., 2016.[6]	GAAAGCAAGCTCGTTTACGG	AGGTTACCAGCCACAACCAG
Wdfy3$^{fl/fl}$: 129/SvEv x C57BL/6 (flanking Exon 6)	Ai Yamamoto Lab, Dragich et al., 2016.[6]	ACTTGGGAAGAGGGAAGCTC	AGGTTACCAGCCACAACCAG
Wdfy3$^{fl/fl}$: C57BL/6NJ (flanking Exon 8)	Konstantinos Zarbalis Lab, Orosco, L. A. et al. 2014.[7]	AGTGCAAATAAAGAACTAAATTAGAAGG	CATAACTTCGTATAATGTATGCTATACG
Wdfy3$^{fl/fl}$: C57BL/6NJ (flanking Exon 8)	Konstantinos Zarbalis Lab, Orosco, L. A. et al. 2014.[7]	ACAGGTCTCTTTGGCTGAGG	AATGTCTTGCGTCGGAAAAG

Supplementary Table 6. Primers for quantitative RT-PCR

Target	Source	Forward (5’ to 3’)	Reverse (5’ to 3’)
Human: *ACTB*	PrimerBank	CATGTACGTTGCTATCCAGGC	CTCCTTAATGTCACGCACGAT
Human: *WDFY3*	OriGene	GACAACCTCTGTCTCACTCCTG	GCAAATGGTCCATCACGCTATCC

Supplementary Table 7. siRNAs

Reagent or Resource	Source	Identifier
ON-TARGET plus non-targeting pool	Dharmacon	D-001810-10-05
ON-TARGET plus non-targeting siRNA #1	Dharmacon	D-001810-01-20
ON-TARGET plus Human WDFY3 (23001) siRNA - SMARTpool	Dharmacon	L-012924-01-0005
ON-TARGET plus Human WDFY3 (23001) siRNA - Individual	Dharmacon	J-012924-09-0010

Supplementary Table 8. Antibodies

Reagents	Source	Identifier	Dilution and Working Concentration
CD16/32 (Purified anti-mouse CD16/32), monoclonal, Rat IgG2a, λ	BioLegend	Cat# 101302 (Reactivity: Mouse)	1:25 (block), 20 µg/mL
Goat anti-rabbit IgG (Fc, HRP), polyclonal, IgG	EMD Millipore	Cat# AP156P (Reactivity: Rabbit)	1:5000 (WB), 0.16 µg/mL
Anti-β-Actin (13E5, HRP), rabbit monoclonal, IgG	Cell Signaling Technology	Cat# 5125S, Lot 6 (48 µg/mL) (Reactivity: Human, Mouse, Rat, Monkey, Bovine, Pig)	1:5000 (WB), 0.0096 µg/mL
Anti-GABARAP (N-term), rabbit polyclonal, IgG	Abgent	Cat# AP1821a (Reactivity: Human, Mouse, Rat)	1:1000 (WB), 0.025 µg/mL
Anti-GABARAP + GABARAPL1 + GABARAPL3 (EPR18862), rabbit monoclonal, IgG	Abcam	Cat# ab191888 (Reactivity: Human, Mouse, Rat)	9 ug/mL (IP)
Anti-LC3A/B (D3U4C, PE), rabbit monoclonal, IgG	Cell Signaling Technology	Cat# 13611S (Reactivity: Human, Mouse, Rat)	1:50 (FACS), 0.5 µg/mL
Anti-LC3A/B (D3U4C, Alexa Fluor 488), rabbit monoclonal, IgG	Cell Signaling Technology	Cat# 13082S (Reactivity: Human, Mouse, Rat)	1:50 (FACS), 1.2 µg/mL
Anti-LC3B, rabbit polyclonal, IgG	Abcam	Cat# ab48394 (Reactivity: Human, Mouse, Rat)	1:1000 (WB), 1 µg/mL
Anti-WDFY3, rabbit monoclonal	Ai Yamamoto Lab, Fox et al., 2020.[10]	Cat# NA (Reactivity: Human, Mouse)	1:1000 (WB)
Anti-CD68 (FA-11), rat monoclonal, IgG2a	Abcam	Cat# ab53444 (Reactivity: Mouse)	1:200 (IF), 5 µg/mL
Anti-F4/80 (BM8, FITC), rat monoclonal, IgG2a, κ	BioLegend	Cat# 123108 (Reactivity: Mouse)	1:200 (FACS), 2.5 µg/mL
Anti-F4/80 (BM8, APC-Cy7), rat monoclonal, IgG2a, κ	BioLegend	Cat# 123118 (Reactivity: Mouse)	1:200 (FACS), 1 µg/mL

Supplementary Table 9. Cell Culture Medium

Reagent or Resource	Source	Identifier
CellStripper	Corning	Cat# 25-056-CI
Dulbecco’s Modified Eagle Media (DMEM)	Corning	Cat# 10-017-CM
Dulbecco's Phosphate-Buffered Salt Solution 1X (DPBS)	Corning	Cat# 21-031-CM
Heat-Inactivated Fetal Bovine Serum	Gibco	Cat# 10082147
Opti-MEM I Reduced Serum Medium	Gibco	Cat# 31985070
Roswell Park Memorial Institute (RPMI) 1640 Media	Corning	Cat# 10-041-CM

Supplementary Table 10. Chemicals and Recombinant Cytokines

Reagent or Resource	Source	Identifier
Cytochalasin D	Sigma-Aldrich	Cat# C8273
Dexamethasone	Sigma-Aldrich	Cat# 265005-100MG
Digitonin	Sigma-Aldrich	Cat# D141-500MG
Human Macrophage Colony Stimulating Factor (M-CSF)	Goldbio	Cat# 1120-09-100
LY294002	Sigma-Aldrich	Cat# 440204-1MG
Puromycin	Sigma-Aldrich	Cat# 540411-25MG
Staurosporine	Alfa Aesar	Cat# J62837-M^

Supplementary Table 11. Commercial Assay Kits

Reagent or Resource	Source	Identifier
BCA Protein Assay Kit	Thermo Scientific	Cat# 23227
DNeasy Blood and Tissue Kit	Qiagen	Cat# 69504
High-Capacity cDNA Reverse Transcription Kit	Applied Biosystems	Cat# 4368814
In Situ Cell Death Detection Kit, TMR red (TUNEL)	Roche	Cat# 12156792910
Quick-RNA Mini Kit	Zymo	Cat# R1055
West Pico PLUS Chemiluminescent Substrate	Thermo Scientific	Cat# 34580

Supplementary Table 12. Reagents for Efferocytosis and Phagocytosis Assays

Reagent or Resource	Source	Identifier
Annexin V Conjugates for Apoptosis Detection	Invitrogen	Cat# A23204
CellTracker Green CMFDA Dye	Invitrogen	Cat# C2925
CellTracker Deep Red Dye	Invitrogen	Cat# C34565
CellMask Deep Red Actin Tracking Stain	Invitrogen	Cat# A57245
CellMask Green Actin Tracking Stain	Invitrogen	Cat# A57243
Diluent C	Sigma-Aldrich	Cat# CGLDIL-6X10ML
FluoSpheres Sulfate Microspheres, 4.0 μm, red fluorescent (580/605)	Invitrogen	Cat# F8858
FluoSpheres Polystyrene Microspheres, 10 μm, blue-green fluorescent (430/465)	Invitrogen	Cat# F8830
FluoSpheres Polystyrene Microspheres, 10 μm, orange fluorescent (540/560)	Invitrogen	Cat# F8833
FluoSpheres Polystyrene Microspheres, 10 μm, red fluorescent (580/605)	Invitrogen	Cat# F8834
HCS NuclearMask™ Blue Stain	Invitrogen	Cat# H10325
Hoechst 33342 Solution	Thermo Scientific	Cat# 62249
PBS (1X)	Corning	Cat# 21-040-CV
PBS (10X)	Corning	Cat# 46-013-CM
pHrodo Red, succinimidyl ester	Invitrogen	Cat# P36600
PKH26 Red Fluorescent Cell Linker Kit for General Cell Membrane Labeling	Sigma-Aldrich	Cat# PKH26GL-1KT
PKH67 Green Fluorescent Cell Linker Kit for General Cell Membrane Labeling	Sigma-Aldrich	Cat# PKH67GL-1KT
Sheep Red Blood Cells 10% washed pooled cells	Rockland Immunochemicals	Cat# R405-0050
Sheep Red Blood Cell RBC Antibody	Rockland Immunochemicals	Cat# 213-4139
siR-actin	Cytoskeleton	Cat# CY-SC001
TAMRA, SE (5-(and-6)-Carboxytetramethylrhodamine, Succinimidyl Ester (5(6)-TAMRA, SE), mixed isomers)	Invitrogen	Cat# C1171
Zymosan A (S. cerevisiae) BioParticles, Alexa Fluor 594 conjugate	Invitrogen	Cat# Z23374

Supplementary Table 13. Other Reagents and Supplies

Reagent or Resource	Source	Identifier
Ficoll-Paque Premium	Sigma-Aldrich	Cat# GE17-5442-02
Pierce 16% Formaldehyde (w/v), Methanol-free	Thermo Scientific	Cat# 28908
Fugene 6	Promega	Cat# E2691
Immun-Blot PVDF Membrane, 0.2 µm	Bio-rad	Cat# 1620177
Lenti-X Concentrator	Takara Bio	Cat# 631231
Lipofectamine RNAiMAX Transfection Reagent	Invitrogen	Cat# 13778100
µ-Slide 8 Well	Ibidi	Cat# 80826
NuPAGE 3 to 8%, Tris-Acetate, 1.5 mm, Mini Protein Gel	Invitrogen	Cat# EA0378BOX
Novex Bolt LDS Sample Buffer (4X)	invitrogen	Cat# B0007
Novex Bolt Sample Reducing Agent (10X)	Invitrogen	Cat# B0009
Novex WedgeWel 16%, Tris-Glycine, 1.0 mm, Mini Protein Gels	Invitrogen	Cat# XP00165BOX
P3 Primary Cell 4D-Nucleofector™ X Kit S	Lonza	Cat# V4XP-3032
Power SYBR Green PCR Master Mix	Applied Biosystems	Cat# 4367659
Protease Inhibitor Cocktail	Roche	Cat# 11697498001
Protein A/G Agarose Beads	Thermo Scientific	Cat# 20421
PVDF Transfer Membrane, 0.45 µm	Thermo Scientific	Cat# 88518
RIPA Lysis Buffer, 10X	Millipore Sigma	Cat# 20-188

Supplementary Table 14. Software and Algorithms

Reagent or Resource	Source	Identifier
BD FACSDiva	BD Biosciences	V9.0
DESeq2	Love et al., 2018.[11]	https://bioconductor.org/packages/release/bioc/html/DESeq2.html<br>v1.38.1
FCS Express	De Novo Software	Research Version 7
FIJI	NIH	https://fiji.sc/<br>v2.3.0 Java 1.8.0_202
GSEA	Subramanian et al., 2005.[12]	Version 4.2.0
ImageJ JACoP	NIH and Bolte et al., 2006.[13]	ImageJ bundled with 64-bit Java 1.8.0_172<br>JACoP (Just Another Colocalization Plugin[13] version 2.0)
Ingenuity Pathway Analysis (IPA)	Qiagen	2022 License
MAGeCK	Li et al., 2014.[14]	https://sourceforge.net/p/mageck/wiki/Home/<br>v0.5.7
MetaXpress	Molecular Devices	Version 6
NIS-Elements	Nikon	Version 5.11
PRISM	GraphPad Software	Version 7
Salmon	Patro et al., 2017.[15]	https://combine-lab.github.io/salmon/<br>v1.5.1
tximport	Soneson et al., 2015.[16]	https://bioconductor.org/packages/release/bioc/html/tximport.html<br>v1.26.0

Supplementary Notes

Supplementary Note 1

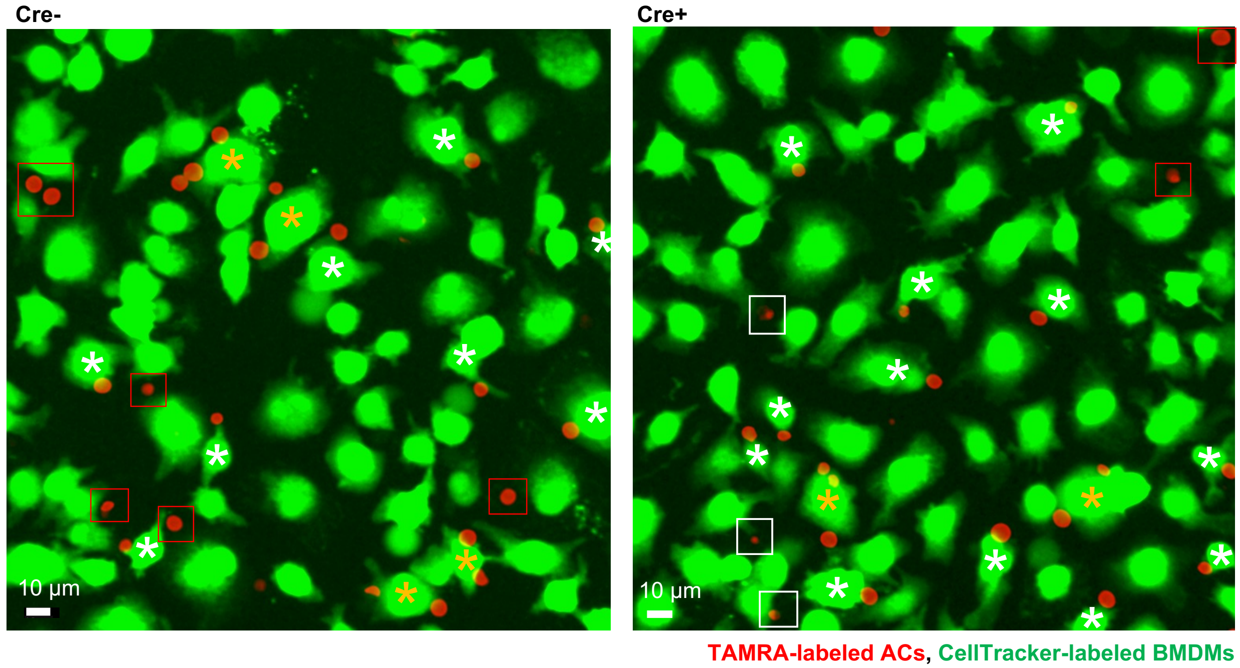

Supplementary Note 1 Quantification of binding assay.

- **The goal of the quantification:**
  Score the macrophages with no AC bound (0), with one AC bound (1), and with two or more than two ACs bound (>2), as shown in **Fig. 2e**.
- **The labeling:**
  The CellTracker Green CMFDA fluorescent dye has been designed to freely pass through cell membranes into cells, where it is transformed into a cell-impermeant, fluorescent product, and used for labeling macrophages. ACs were labeled by TAMRA that stains proteins and peptides.
- **Image preprocessing:**
  To facilitate the scoring of whether ACs are bound to macrophages on the 2D images, we first adjust brightness and contrast for all images to be quantified to improve visualization of the cell contour (as shown in the representative images above). Extra care was taken not to introduce artifacts, including to overlay the adjusted images with phase-contract images to confirm cell contour can be visualized consistently.
- **Exclusion criteria:**
  Because the images were taken at one focal plane, we only quantify the macrophages and ACs with bright signals because cells at different focal planes showing weaker signals do not allow precise scoring. White square highlights TAMRA signals excluded from the analyses because the small size likely suggests debris.
- **Scoring criteria:**
  The TAMRA-labeled ACs and CellTracker Green-labeled macrophages in close contact are scored as "bound" and counted. In the representative images, white * denotes macrophages with one bound AC (1), orange * denotes macrophages with two or more bound ACs (>2), red squares highlight the ACs not bound to any macrophages. The investigator is blinded from the genotype while performing scoring.

Supplementary Note 2

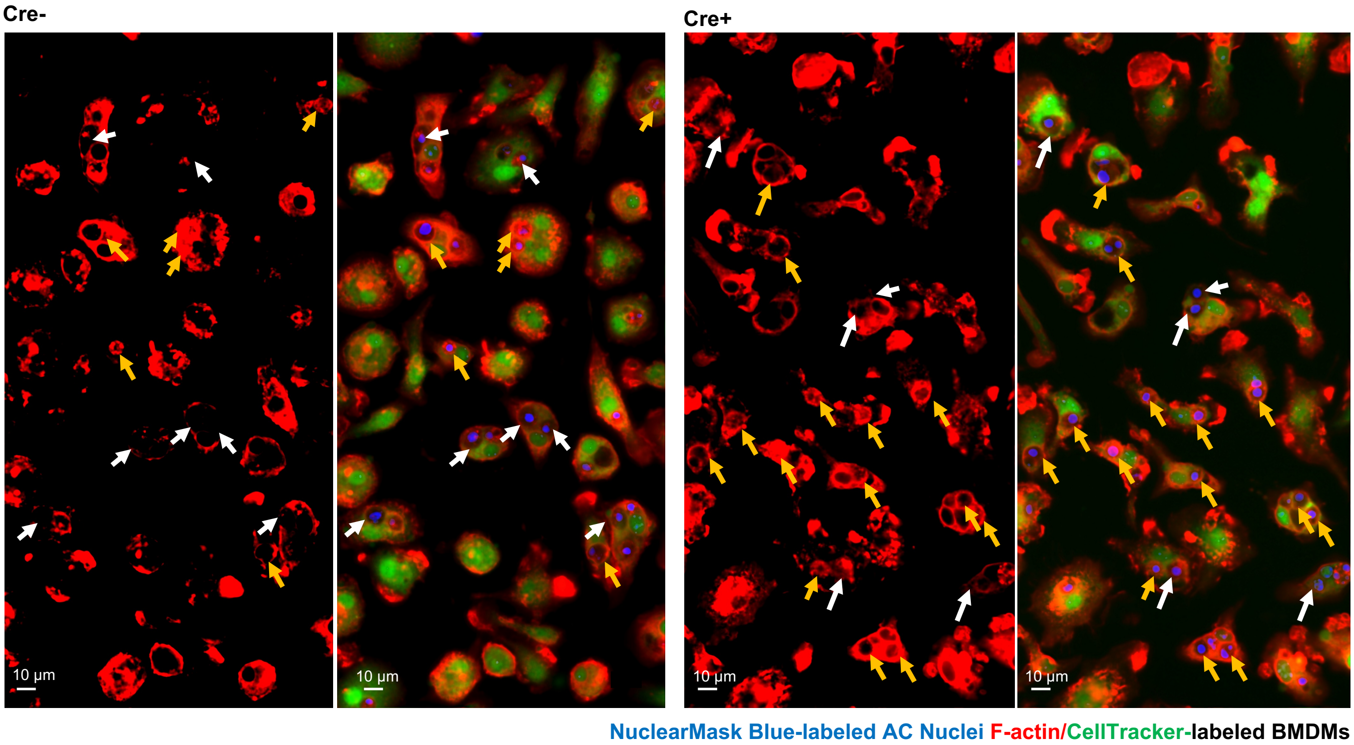

Supplementary Note 2 Quantification of F-actin-ring surrounded engulfed ACs.

- **The goal of the quantification:**
  Score the macrophages with engulfed ACs surrounded by a F-actin ring, as shown in **Fig. 2h**, quantified as the percentage of macrophages with F-actin ring surrounded ACs in the macrophages with engulfed ACs.
- **The labeling:**
  The CellTracker Green CMFDA fluorescent dye labels the macrophages. Macrophages were also labeled with siR-actin, a fluorogenic, cell-permeable probe based on a highly specific F-actin binding natural product jasplakinolide, therefore can visualize F-actin. ACs were labeled by NuclearMask Blue that stains nuclear DNAs.
- **Image preprocessing:**
  F-actin only and the merged images were compared in parallel in order to better determine the presence of "ring"-like morphology of the F-actin staining.
- **Exclusion criteria:**
  Cutoff to size was applied to NuclearMask Blue staining because small size likely suggests debris.
- **Scoring criteria:**
  The first step was to determine if a macrophage has engulfed a NuclearMask Blue-labeled AC. Because the size of the AC is ~10 µm, the NuclearMask Blue-stained nuclei of the engulfed AC and the unstained cytoplasmic area around the AC nuclei can be clearly identified within CellTracker-labeled macrophages. The presence of F-actin ring can be determined by referring both the F-actin only and the merged images. A continued F-actin staining surrounded the engulfed ACs by macrophages is scored as macrophages with engulfed AC with F-actin ring and denoted by orange arrows. An absence or discontinued F-actin staining is scored as macrophages without F-actin ring and denoted by white arrows. The investigator is blinded from the genotype while performing scoring.

Supplementary Note 3

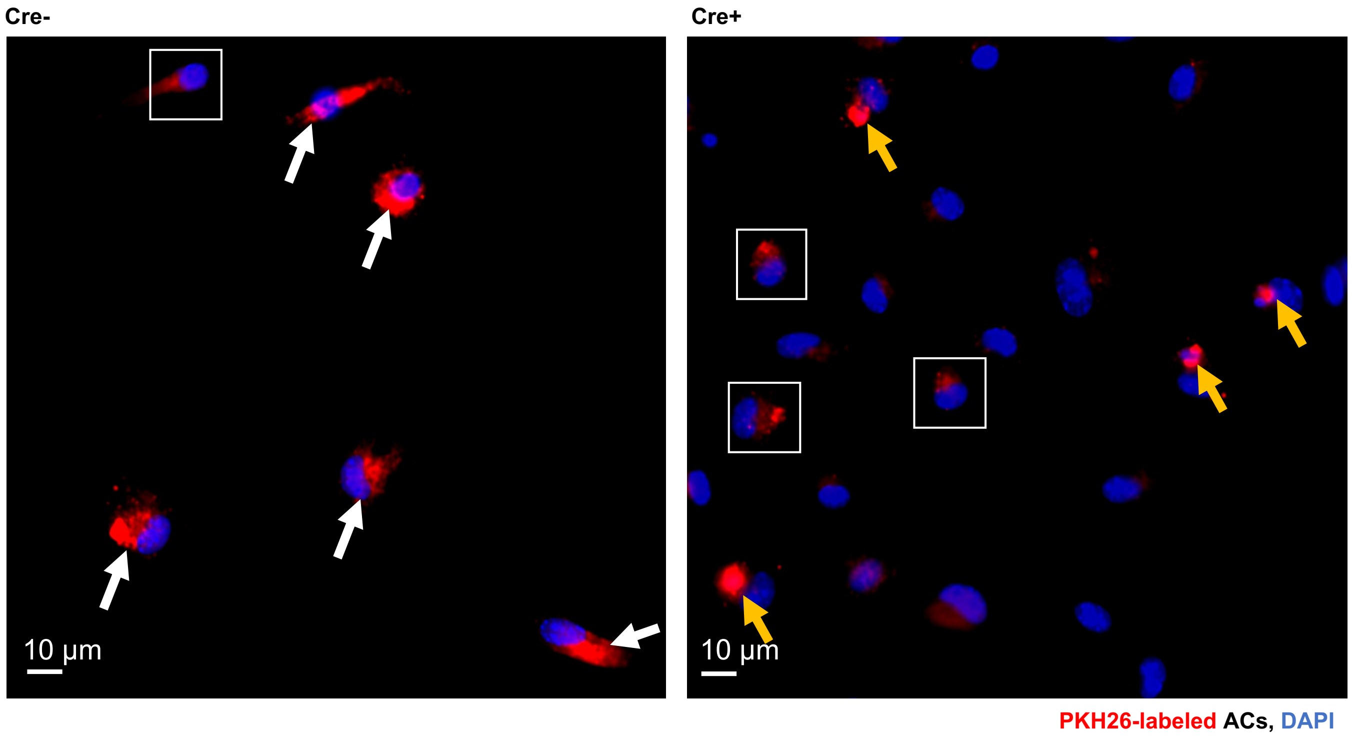

Supplementary Note 3 Quantification of non-fragmented ACs (for BMDM and HMDM)

- **The goal of the quantification:**
  Score the macrophages with non-fragmented engulfed ACs, implying impaired degradation, as shown in **Fig. 3a** (and **Fig. 6e)**, quantified as the ratio of the number of macrophages with non-fragmented ACs by the total number of macrophages with engulfed ACs.
- **The labeling:**
  ACs were labeled by PKH26 red fluorescence dye. The PKH lipophilic dyes are highly fluorescent and stain membranes by intercalating their aliphatic portion into the exposed lipid bilayer[17]. DAPI was used to stain nuclear DNA (in both ACs and macrophages).
- **Image preprocessing:**
  N/A.
- **Exclusion criteria:**
  Macrophages with weak and pixelated red signals without an visible AC nuclei stained by DAPI are macrophages that engulfed cell debris, as denoted by white squares. Those macrophages were not scored as macrophage with engulfed ACs.
- **Scoring criteria:**
  Macrophages with PKH26 signal in a circular shape without obvious pattern of fragments were scored as “non-fragmented” and denoted by orange arrows. Macrophages with PKH26 signal in an irregular shape and a fragmented pattern were scored as “fragmented”. The investigator is blinded from the genotype while performing scoring.

Supplementary References

1 Haney, M. S. *et al.* Identification of phagocytosis regulators using magnetic genome-wide CRISPR screens. *Nature genetics* **50**, 1716-1727, doi:10.1038/s41588-018-0254-1 (2018).

2 Kamber, R. A. *et al.* Inter-cellular CRISPR screens reveal regulators of cancer cell phagocytosis. *Nature* **597**, 549-554, doi:10.1038/s41586-021-03879-4 (2021).

3 Monaco, G. *et al.* RNA-Seq Signatures Normalized by mRNA Abundance Allow Absolute Deconvolution of Human Immune Cell Types. *Cell reports* **26**, 1627-1640.e1627, doi:10.1016/j.celrep.2019.01.041 (2019).

4 Eenjes, E., Dragich, J. M., Kampinga, H. H. & Yamamoto, A. Distinguishing aggregate formation and aggregate clearance using cell-based assays. *Journal of cell science* **129**, 1260-1270, doi:10.1242/jcs.179978 (2016).

5 Kuma, A. & Mizushima, N. Chromosomal mapping of the GFP-LC3 transgene in GFP-LC3 mice. *Autophagy* **4**, 61-62, doi:10.4161/auto.4846 (2008).

6 Dragich, J. M. *et al.* Autophagy linked FYVE (Alfy/WDFY3) is required for establishing neuronal connectivity in the mammalian brain. *eLife* **5**, doi:10.7554/eLife.14810 (2016).

7 Orosco, L. A. *et al.* Loss of Wdfy3 in mice alters cerebral cortical neurogenesis reflecting aspects of the autism pathology. *Nature communications* **5**, 4692, doi:10.1038/ncomms5692 (2014).

8 Filimonenko, M. *et al.* The selective macroautophagic degradation of aggregated proteins requires the PI3P-binding protein Alfy. *Molecular cell* **38**, 265-279, doi:10.1016/j.molcel.2010.04.007 (2010).

9 Fang, J. *et al.* Metformin alleviates human cellular aging by upregulating the endoplasmic reticulum glutathione peroxidase 7. *Aging Cell* **17**, e12765, doi:10.1111/acel.12765 (2018).

10 Fox, L. M. *et al.* Huntington's Disease Pathogenesis Is Modified In Vivo by Alfy/Wdfy3 and Selective Macroautophagy. *Neuron* **105**, 813-821.e816, doi:10.1016/j.neuron.2019.12.003 (2020).

11 Love, M. I., Soneson, C. & Patro, R. Swimming downstream: statistical analysis of differential transcript usage following Salmon quantification. *F1000Res* **7**, 952, doi:10.12688/f1000research.15398.3 (2018).

12 Subramanian, A. *et al.* Gene set enrichment analysis: a knowledge-based approach for interpreting genome-wide expression profiles. *Proceedings of the National Academy of Sciences of the United States of America* **102**, 15545-15550, doi:10.1073/pnas.0506580102 (2005).

13 Bolte, S. & Cordelières, F. P. A guided tour into subcellular colocalization analysis in light microscopy. *J Microsc* **224**, 213-232, doi:10.1111/j.1365-2818.2006.01706.x (2006).

14 Li, W. *et al.* MAGeCK enables robust identification of essential genes from genome-scale CRISPR/Cas9 knockout screens. *Genome biology* **15**, 554, doi:10.1186/s13059-014-0554-4 (2014).

15 Patro, R., Duggal, G., Love, M. I., Irizarry, R. A. & Kingsford, C. Salmon provides fast and bias-aware quantification of transcript expression. *Nature methods* **14**, 417-419, doi:10.1038/nmeth.4197 (2017).

16 Soneson, C., Love, M. I. & Robinson, M. D. Differential analyses for RNA-seq: transcript-level estimates improve gene-level inferences. *F1000Res* **4**, 1521, doi:10.12688/f1000research.7563.2 (2015).

17 Pužar Dominkuš, P. *et al.* PKH26 labeling of extracellular vesicles: Characterization and cellular internalization of contaminating PKH26 nanoparticles. *Biochim Biophys Acta Biomembr* **1860**, 1350-1361, doi:10.1016/j.bbamem.2018.03.013 (2018).
