# Supplementary material for: A genome-wide CRISPR screen identifies WDFY3 as a regulator of macrophage efferocytosis
Source: Nat Commun. 2022 Dec 24;13:7929. doi: 10.1038/s41467-022-35604-8 (PMC9789999; doi:10.1038/s41467-022-35604-8)
Supplement: Supplementary file 3 — Description of Additional Supplementary Files [file 41467_2022_35604_MOESM3_ESM.pdf]

Description of Additional Supplementary Files

A Genome-wide CRISPR Screen Identifies WDFY3 as a Regulator of Macrophage Efferocytosis

Jianting Shi, Xun Wu et al.

Supplementary Data

Supplementary Data 1:
MAGeCK Analysis of CRISPR Screening of Macrophage Efferocytosis: Input vs. Non-eaters

Supplementary Data 2:
MAGeCK Analysis of CRISPR Screening of Macrophage Efferocytosis: Input vs. Efficient Eaters

Supplementary Data 3:
MAGeCK Analysis of CRISPR Screening of Macrophage Efferocytosis: Non-eaters vs. Efficient Eaters

Supplementary Data 4:
Canonical Pathway Analysis of Positive Regulators by Ingenuity Pathway Analysis (IPA)

Supplementary Data 5:
Canonical Pathway Analysis of Negative Regulators by IPA

Supplementary Data 6:
Comparing the Top-ranked Positive Regulators by Our Screen and Previous Screens in Monocytic Cell Line-derived Macrophages

Supplementary Data 7:
DESeq2 Results of RNA-seq of Cre^- and Cre^+ BMDMs

Supplementary Data 8:
GSEA Output of Human Reactome Pathway Enriched in Upregulated Genes in Cre^+ BMDMs

Supplementary Data 9:
GSEA Output of Gene Ontology Biological Process Terms Enriched in Upregulated Genes in Cre^+ BMDMs

Supplementary Data 10:
GSEA Output of Human Reactome Pathway Enriched in Downregulated Genes in Cre^+ BMDMs

Supplementary Data 11:
GSEA Output of Gene Ontology Biological Process Terms Enriched in Downregulated Genes in Cre^+ BMDMs

Supplementary Movies

Supplementary Movie 1: Synchronized F-actin polymerization and depolymerization during efferocytosis.

Bone-marrow-derived macrophages (BMDMs) from wild-type C57BL/6J mice were transfected with LifeAct-tagGFP2 mRNA encoding a fusion protein of LifeAct and a GFP reporter for visualization of cellular F-actin. The transfected BMDMs were then stained with CellTracker Deep Red, a fluorescent dye that freely passes through cell membranes and is well-retained in cells, allowing labeling of cytoplasmic area and tracking cell movements. BMDMs undergoing efferocytosis of unlabeled apoptotic Jurkat cells were imaged with a Nikon Ti Eclipse inverted microscope for spinning-disk confocal microscopy equipped with a 100x/1.49 Apo TIRF oil immersion lens. Time-lapse images of the same fields were acquired at 30 s intervals for 20 min, and the movie was prepared and annotated using FIJI v2.3.0. As pointed out by the white arrow, the phagocytic cup formation is visualized by emerged F-actin signals, followed by engulfment of the apoptotic cell initially surrounded by F-actin. The complete engulfment is synchronized by reduced F-actin signals, indicating depolymerization.

Supplementary Movie 2: Z-stack imaging of engulfed beads surrounded by F-actin rings.

Z-stack imaging was performed in order to visualize and determine if engulfed beads by macrophages were surrounded by F-actin rings. This reconstructed video serves as an example of F-actin ring-surrounded beads. Macrophages were stained with CellMask Deep Red Actin Tracking Stain and incubated with 10 μm blue fluorescent (Ex365/Em415) polystyrene beads for 15 min and then fixed. Z-stack imaging was performed using a Nikon Ti Eclipse inverted microscope for spinning-disk confocal microscopy equipped with a 100x/1.49 Apo TIRF oil immersion lens. Images in a z-series through the cells were acquired at 0.5 μm intervals and reconstructed into a video that visualizes F-actin ring-surrounded beads at different focal planes as denoted by the white arrows. FIJI v2.3.0 was used to process and annotate the movie.
