# Supplementary material for: A genome-wide CRISPR screen identifies WDFY3 as a regulator of macrophage efferocytosis
Source: Nat Commun. 2022 Dec 24;13:7929. doi: 10.1038/s41467-022-35604-8 (PMC9789999; doi:10.1038/s41467-022-35604-8)
Supplement: Supplementary file 17 — Reporting Summary [file 41467_2022_35604_MOESM17_ESM.pdf]

nature portfolio

Corresponding author(s): Hanrui Zhang

Last updated by author(s): Nov 30, 2022

Reporting Summary

Nature Portfolio wishes to improve the reproducibility of the work that we publish. This form provides structure for consistency and transparency in reporting. For further information on Nature Portfolio policies, see our Editorial Policies and the Editorial Policy Checklist.

Statistics

For all statistical analyses, confirm that the following items are present in the figure legend, table legend, main text, or Methods section.

n/a	Confirmed	
☐	☒	The exact sample size (*n*) for each experimental group/condition, given as a discrete number and unit of measurement
☐	☒	A statement on whether measurements were taken from distinct samples or whether the same sample was measured repeatedly
☐	☒	The statistical test(s) used AND whether they are one- or two-sided<br>*Only common tests should be described solely by name; describe more complex techniques in the Methods section.*
☒	☐	A description of all covariates tested
☒	☐	A description of any assumptions or corrections, such as tests of normality and adjustment for multiple comparisons
☐	☒	A full description of the statistical parameters including central tendency (e.g. means) or other basic estimates (e.g. regression coefficient) AND variation (e.g. standard deviation) or associated estimates of uncertainty (e.g. confidence intervals)
☐	☒	For null hypothesis testing, the test statistic (e.g. *F*, *t*, *r*) with confidence intervals, effect sizes, degrees of freedom and *P* value noted<br>*Give P values as exact values whenever suitable.*
☒	☐	For Bayesian analysis, information on the choice of priors and Markov chain Monte Carlo settings
☒	☐	For hierarchical and complex designs, identification of the appropriate level for tests and full reporting of outcomes
☐	☒	Estimates of effect sizes (e.g. Cohen's *d*, Pearson's *r*), indicating how they were calculated

Our web collection on statistics for biologists contains articles on many of the points above.

Software and code

Policy information about availability of computer code

Data collection	BD FACSDiva v9.0 was used to collect data from BD LSRFortessa. MetaXpress Version 6 was used to collect data from ImageXpress Micro4; NIS-Elements Version 5.11 was used to collect images from Nikon Ti Eclipse inverted microscope;
Data analysis	DESeq2 v1.38.1; FCS Express Research Version 7; FIJI v2.3.0 Java 1.8.0_202; GSEA 4.2.0; ImageJ bundled with 64-bit Java 1.8.0_172 and JACoP (Just Another Colocalization Plugin13 version 2.0); Ingenuity Pathway Analysis (IPA) 2022 License; MAGeCK v0.5.7; Prism Version 7; Salmon v1.5.1; tximport.v1.26.0.<br><br>Code used for data analyses is available via the Zhang lab GitHub repository https://github.com/hanruizhang/NatCommun-bioRxiv.2022.477299.

For manuscripts utilizing custom algorithms or software that are central to the research but not yet described in published literature, software must be made available to editors and reviewers. We strongly encourage code deposition in a community repository (e.g. GitHub). See the Nature Portfolio guidelines for submitting code & software for further information.

Data

Policy information about availability of data

All manuscripts must include a data availability statement. This statement should provide the following information, where applicable:

- Accession codes, unique identifiers, or web links for publicly available datasets
- A description of any restrictions on data availability
- For clinical datasets or third party data, please ensure that the statement adheres to our policy

The datasets generated in this study have been deposited in the Gene Expression Omnibus (GEO), including RNA-seq datasets under accession code GSE211694 (https://www.ncbi.nlm.nih.gov/geo/query/acc.cgi?acc=GSE211694) and CRISPR screening datasets under accession code GSE212008 (https://www.ncbi.nlm.nih.gov/geo/query/acc.cgi?acc=GSE212008). The human macrophage RNA-seq dataset was previously published and are available at DRYAD with identifier doi:10.5061/dryad.866t1g1nb. Source data are provided in the Supplementary Information/Supplementary Data/Source Data file.

Human research participants

Policy information about studies involving human research participants and Sex and Gender in Research.

Reporting on sex and gender

Buffy coats of anonymous, de-identified healthy adult volunteer donors were purchased from the New York Blood Center (NYBC), with informed consent obtained by the NYBC.

Because the buffy coats were purchased from commercial sources, anonymous and de-identified, and we do not need to recruit subjects, the research was determined to be “Not Human Subjects Research” upon review by the Institutional Review Board at Columbia University.

Population characteristics

NA

Recruitment

NA

Ethics oversight

NA

Note that full information on the approval of the study protocol must also be provided in the manuscript.

Field-specific reporting

Please select the one below that is the best fit for your research. If you are not sure, read the appropriate sections before making your selection.

☒ Life sciences ☐ Behavioural & social sciences ☐ Ecological, evolutionary & environmental sciences

For a reference copy of the document with all sections, see nature.com/documents/nr-reporting-summary-flat.pdf

Life sciences study design

All studies must disclose on these points even when the disclosure is negative.

Sample size

No statistical methods were used to predetermine sample size.

Sample size for cell-based experiments at 3-6 independent experiments/biological replicates were determined based on literature describing in vitro efferocytosis assays (e.g. Cell. 2017 Oct 5;171(2):331-345.e22. Cell Metab. 2020 Mar 3;31(3):518-533.e10. J Clin Invest. 2021 Apr 15;131(8):e145275. Nat Metab. 2022 Apr;4(4):444-457.) Based on our data in Fig. 2c (n=3) and Supplementary Fig. 5a (n=3-5) determining how knockout of Wdfy3 impacts efferocytosis in BMDMs, a n= 3 or 2 per group, respectively, was sufficient to achieve 0.8 power to detect differences at a significance level of 0.05 (calculated using means and pooled SD in powerandsamplesize.com with 2-samples 2-sided equality).

Sample size for mouse experiments were determined empirically according to the above literature performing in vivo efferocytosis. Based on our data in Fig. 5f (n=5) and 5h (n=5) determining how knockout of Wdfy3 impacts efferocytosis in vivo (thymus and peritoneum, respectively), a n=4 per group was sufficient to achieve 0.8 power to detect differences at a significance level of 0.05 (calculated using means and pooled variance in powerandsamplesize.com with 2-samples 2-sided equality).

Data exclusions

One out of the three screening replicates was excluded because the flow sorter was clogged during sorting that took hours to resolve. We suspect the clogging and delay might have negatively affected cell viability and sorting accuracy, and have excluded the data for the analysis.

Replication

Each experiment was repeated and the number of independent experiments or technical replicates was described in the respective Figure Legends. All attempts were successful at replication although there were some variations of values.

Randomization	Randomization was not applicable as the groups were determined by genotype.
Blinding	For CBC measurement and RNA-seq library preparation, the technicians were blinded from experimental design. Imaging data were also analyzed blindly. For other experiments, investigators were not blinded to mouse genotypes during data collection and analysis to ensure that age/sex-matched pairs were used for each genotype and in each independent experiment.

Reporting for specific materials, systems and methods

We require information from authors about some types of materials, experimental systems and methods used in many studies. Here, indicate whether each material, system or method listed is relevant to your study. If you are not sure if a list item applies to your research, read the appropriate section before selecting a response.

Materials & experimental systems

n/a	Involved in the study
☐	☒ Antibodies
☐	☒ Eukaryotic cell lines
☒	☐ Palaeontology and archaeology
☐	☒ Animals and other organisms
☒	☐ Clinical data
☒	☐ Dual use research of concern

Methods

n/a	Involved in the study
☒	☐ ChIP-seq
☐	☒ Flow cytometry
☒	☐ MRI-based neuroimaging

Antibodies

Antibodies used

Supplementary Table 8: Reagents, Source, Identifier, Dilution and Working Concentration.

CD16/32 (Purified anti-mouse CD16/32), monoclonal, Rat IgG2a, λ, BioLegend, Cat# 101302 (Reactivity: Mouse), 1:25 (block), 20 μg/mL.

Goat anti-rabbit IgG (Fc, HRP), polyclonal, IgG, EMD Millipore, Cat# AP156P (Reactivity: Rabbit), 1:5000 (WB), 0.16 μg/mL.

Anti-β-Actin (13E5, HRP), rabbit monoclonal, IgG, Cell Signaling Technology, Cat# 5125S, Lot 6 (48 μg/mL) (Reactivity: Human, Mouse, Rat, Monkey, Bovine, Pig), 1:5000 (WB), 0.0096 μg/mL.

Anti-GABARAP (N-term), rabbit polyclonal, IgG, Abgent, Cat# AP1821a (Reactivity: Human, Mouse, Rat), 1:1000 (WB), 0.025 μg/mL.

Anti-GABARAP + GABARAPL1 + GABARAPL3 (EPR18862), rabbit monoclonal, IgG, Abcam, Cat# ab191888 (Reactivity: Human, Mouse, Rat, 9 ug/mL (IP).

Anti-LC3A/B (D3U4C, PE), rabbit monoclonal, IgG Cell Signaling Technology, Cat# 13611S
(Reactivity: Human, Mouse, Rat), 1:50 (FACS), 0.5 μg/mL.

Anti-LC3A/B (D3U4C, Alexa Fluor 488), rabbit monoclonal, IgG, Cell Signaling Technology, Cat# 13082S (Reactivity: Human, Mouse, Rat), 1:50 (FACS), 1.2 μg/mL.

Anti-LC3B, rabbit polyclonal, IgG, Abcam, Cat# ab48394 (Reactivity: Human, Mouse, Rat), 1:1000 (WB), 1 μg/mL.

Anti-WDFY3, rabbit monoclonal, Ai Yamamoto Lab, Fox et al., 2020, Cat# N/A (Reactivity: Human, Mouse), 1:1000 (WB).

Anti-CD68 (FA-11), rat monoclonal, IgG2a, Abcam, Cat# ab53444 (Reactivity: Mouse), 1:200 (IF), 5 μg/mL.

Anti-F4/80 (BM8, FITC), rat monoclonal, IgG2a, κ, BioLegend, Cat# 123108 (Reactivity: Mouse), 1:200 (FACS), 2.5 μg/mL.

Anti-F4/80 (BM8, APC-Cy7), rat monoclonal, IgG2a, κ, BioLegend, Cat# 123118 (Reactivity: Mouse), 1:200 (FACS), 1 μg/mL.

Validation

The ati-WDFY3 antibody was made and provided by the Ai Yamamoto Lab according to Fox et al 2020. Validation was performed by using macrophages from wild-type and Wdfy3 knockout mice to show the absence of band in the knockout cells (Fig. 2a, Supplementary Fig. 6a, Supplementary Fig. 7a) and human macrophages with siRNA-mediated knockdown of WDFY3 (Fig. 6c).

For other commercial antibodies:
CD16/32 (Purified anti-mouse CD16/32), monoclonal, Rat IgG2a, λ, BioLegend, Cat# 101302: Validated by flow cytometry for successful blocking.

Goat anti-rabbit IgG (Fc, HRP), polyclonal, IgG, EMD Millipore, Cat# AP156P: HRP conjugate is validated for use in ELISA, WB for the

detection of Rabbit IgG. Based on immunoelectrophoresis, the antibody reacts with the heavy chains on rabbit IgG but not with the light chains on most rabbit immunoglobulins. No antibody was detected against rabbit IgM, or against immunoglobulin serum proteins, but antibodies may cross-react with immunoglobulins from other species.

Anti-β-Actin (13E5, HRP), rabbit monoclonal, IgG, Cell Signaling Technology, Cat# 5125S: β-Actin (13E5) Rabbit mAb (HRP Conjugate) detects endogenous levels of total β-actin protein. Despite the high sequence identity between the cytoplasmic actin isoforms, β-actin and cytoplasmic γ-actin, β-Actin (13E5) Rabbit mAb (HRP Conjugate) #5125 does not cross-react with cytoplasmic γ-actin, or any other actin isoforms.

Anti-GABARAP (N-term), rabbit polyclonal, IgG, Abgent, Cat# AP1821a: Citations include PMIDs 32009292, 31519908, 31053714, 30917996, 30783186, 30610711, 31359387.

Anti-GABARAP + GABARAPL1 + GABARAPL3 (EPR18862), rabbit monoclonal, IgG, Abcam, Cat# ab191888: Validated for WB of recombinant human GABARAPL2 full length protein, recombinant human GABARAPL3 full length protein, recombinant human GABARAP full length protein.

Anti-LC3A/B (D3U4C, PE), rabbit monoclonal, IgG Cell Signaling Technology, Cat# 13611S: Validated by flow cytometric analysis of HeLa cells, untreated or treated with chloroquine (50 µM, 16 hr), using LC3A/B (D3U4C) XP® Rabbit mAb (PE Conjugate).

Anti-LC3A/B (D3U4C, Alexa Fluor 488), rabbit monoclonal, IgG, Cell Signaling Technology, Cat# 13082S: Confocal immunofluorescent analysis of HeLa cells, chloroquine-treated (50 µM, overnight), nutrient-starved with EBSS (4 hr), or untreated using LC3A/B (D3U4C) XP®Rabbit mAb (Alexa Fluor® 488 Conjugate) #13082 (green) and β-Actin (8H10D10) Mouse mAb #3700 (red).

Anti-LC3B, rabbit polyclonal, IgG, Abcam, Cat# ab48394: ab48394 has been referenced in 500 publications according to the product webpage.

Anti-CD68 (FA-11), rat monoclonal, IgG2a, Abcam, Cat# ab53444: ab53444 has been referenced in 205 publications according to the product webpage.

Anti-F4/80 (BM8, FITC), rat monoclonal, IgG2a, κ, BioLegend, Cat# 123108: Thioglycolate-elicited Balb/c mouse peritoneal macrophages stained with BM8 FITC. The product has been referenced in 126 publications according to the product webpage.

Anti-F4/80 (BM8, APC-Cy7), rat monoclonal, IgG2a, κ, BioLegend, Cat# 123118: Thioglycolate-elicited Balb/c mouse peritoneal macrophages were stained with CD11b FITC and F4/80 (clone BM8) APC/Cyanine7 or Rat IgG2a, κ APC/Cyanine7 isotype control. The product has been referenced in 66 publications according to the product webpage.

Eukaryotic cell lines

Policy information about cell lines and Sex and Gender in Research

Cell line source(s)

Supplementary Table 1. Cell Lines and Primary Cells
Reagent or Resource, Source, Identifier;
Human: Jurkat cells, ATCC, TIB-152;
Human: THP-1 cells, ATCC, TIB-202;
Human: U937 cells, ATCC, CRL-1593.2;
Human: Peripheral Blood Mononuclear Cells, New York Blood Center, N/A;
Mouse: L-929 Fibroblasts, ATCC, CCL-1;
Mouse: Bone Marrow-Derived Macrophages , This paper, N/A;
Mouse: Peritoneal Macrophages, This paper, N/A.

Authentication

Human Jurkat cells, THP-1, U937 were authenticated by ATCC. The morphology of all cell lines and primary cells were monitored via microscopic examination or stained with antibodies for cell lineage marker by Flow Cytometry for authentication.

Mycoplasma contamination

The Mycoplasma test is regularly performed and each cell line used in the study is negative for Mycoplasma.

Commonly misidentified lines
(See ICLAC register)

No misidentified cell lines were used.

Animals and other research organisms

Policy information about studies involving animals; ARRIVE guidelines recommended for reporting animal research, and Sex and Gender in Research

Laboratory animals

Supplementary Table 2. Mice
Reagent or Resource, Source, Identifier;
Wild-type: C57BL/6J, The Jackson Laboratory, JAX: 000664;
GFP-LC3: C57BL/6J, Ai Yamamoto Lab, Eenjes et al., 2016, Kuma et al., 2008, N/A;

	LysMCre+/-: C57BL/6J, The Jackson Laboratory, JAX: 004781;<br>Rosa-Cas9 knockin: C57BL/6J, The Jackson Laboratory, JAX: 026179;<br>Wdfy3fl/fl: 129/SvEv x C57BL/6 (flanking Exon 6), Ai Yamamoto Lab, Dragich et al., 2016, N/A;<br>Wdfy3fl/fl: C57BL/6NJ (flanking Exon 8), Konstantinos Zarbalis Lab, Orosco, L. A. et al. 2014, N/A.<br><br>Housing conditions: Mice were socially housed in standard cages at 22 °C with 40-60% humidity under a 12-12 h light-dark cycle with ad libitum access to water and food provided by the mouse barrier facility (PicoLab Rodent Diet 20 5053 and 5058, LabDiet).
Wild animals	The study did not involve wild animals.
Reporting on sex	Both male and female mice were used at 8-12 weeks old. We confirmed that Wdfy3 knockout led to impaired efferocytosis in BMDMs from both male and female mice (Supplementary Fig. 5a). Therefore, data from males and females were combined to improve power in this study.<br><br>For RNA-seq, male mice at 9-10 weeks old were used. Because all phenotypic experiments support consistent phenotypes in male and female mice. We consider RNA-seq in male mice should be sufficient to provide molecular insights and for the purpose of hypothesis generation.<br><br>For CRISPR screen, female mice at 8-12 weeks old were used. Because genome-wide screens require a large amount of cells, for practical reasons, screens are typically not performed in both sexes separately. There is also no strong rationale, based on literature, that efferocytosis phenotype is regulated remarkably differently in cells from male and female mice.
Field-collected samples	The study did not involve samples collected from the field.
Ethics oversight	Animal protocols were approved by the Institutional Animal Care And Use Committee at Columbia University (Protocol Number AC-AABN5558)

Note that full information on the approval of the study protocol must also be provided in the manuscript.

Flow Cytometry

Plots

Confirm that:

- [x] The axis labels state the marker and fluorochrome used (e.g. CD4-FITC).
- [x] The axis scales are clearly visible. Include numbers along axes only for bottom left plot of group (a 'group' is an analysis of identical markers).
- [x] All plots are contour plots with outliers or pseudocolor plots.
- [x] A numerical value for number of cells or percentage (with statistics) is provided.

Methodology

Sample preparation	For sample preparation of bone marrow-derived macrophage (BMDM), peritoneal macrophage (PM), and human monocyte-derived macrophage (HMDM) after efferocytosis/phagocytosis assay or LC3 staining for flow cytometry analyses, 1X DPBS was used to gently wash away unbound cargos from the adherent macrophages for at least 3 times. Macrophages were then collected using CellStripper, a non-enzymatic cell dissociation solution, for live cell analysis. The cell suspensions in CellStripper were neutralized with DMEM + 10% HI-FBS (heat-inactivated FBS) and centrifuged to remove the solution. The cell pellets were washed with cold washing buffer (1X DPBS, 2% HI-FBS, 5 mM EDTA, 20 mM HEPES and 1 mM sodium pyruvate) and then resuspended in cold washing buffer for flow cytometry analysis.<br><br>For thymocyte preparation, thymi from mice were dislodged and one lobe of thymus were placed onto a 70 um cell strainer and submerged in cold PBS containing 2% HI-FBS and 1 mM EDTA while being disaggregated into single cells. Thymocytes suspension was then rinsed with PBS containing 2% HI-FBS and 1 mM EDTA and collected for staining of AF647-conjugated Annexin V in Annexin V binding buffer (Invitrogen) at a concentration of 5 × 106 cells/mL for 15 min at room temperature, followed by flow cytometry analysis.<br><br>Mice were injected intraperitoneally with 1×107 TAMRA-stained apoptotic mouse thymocytes in 300 µL PBS. 15min after injection, mice were euthanized and peritoneal exudates were collected. The pelleted cells were blocked with CD16/32 (BioLegend) and then stained by FITC-conjugated F4/80 antibody to label macrophages. PMs were washed in 10 ml cold PBS containing 10% HI-FBS and 2 mM EDTA and centrifuged at 500 x g for 5 min. PMs were resuspended in 200 µL cold PBS containing 2% HI-FBS and 1 mM EDTA and analysis by flow cytometry.
Instrument	BD Influx cell sorter, BD LSRFortessa, BD LSRII
Software	BD FACSDiva 9.0 for data collection; FCS Express Research Version 7 for data analysis.
Cell population abundance	As shown in Supplementary Fig. 8a, bout 95% of macrophages differentiated from isolated bone marrow of mice were F4/80 positive cells; about 90% of peritoneal macrophages prepared from peritoneal exudate ex vivo were F4/80 positive cells.

Gating strategy	For BMDM, PM, and HMDM, FSC-A/SSC-A was used to identify cells of interest and exclude debris that have lower FSC-A/SSC-A. FSC-H/FSC-W or FSC-A/FSC-H was then used to exclude doublets. For efferocytosis/phagocytosis assays, depending on the labeling of the cargo for efferocytosis/phagocytosis (e.g. PKH26/PKH67, Hoechst, pHrodo, or TAMRA), cargo-engulfed macrophages were then gated to determine the % of engulfment or the MFI of the engulfed cargos. For LC3 lipidation, cargo-engulfed macrophages were gated to determine the MFI of LC3-AF488. For LC3 lipidation with GFP-WDFY3 transduction, GFP + macrophages were gated to include macrophages with WDFY3 reconstitution, then cargo-engulfed macrophages were gated to determine the MFI of LC3-PE.<br><br>For in vivo thymus efferocytosis, gating strategy was illustrated in Supplementary Fig. 10a. For in vivo peritoneal efferocytosis, gating strategy was illustrated in Supplementary Fig. 10b.

☒ Tick this box to confirm that a figure exemplifying the gating strategy is provided in the Supplementary Information.
